# Structure sensitivity in the nonscalable regime explored via catalysed ethylene hydrogenation on supported platinum nanoclusters

**DOI:** 10.1038/ncomms10389

**Published:** 2016-01-28

**Authors:** Andrew S. Crampton, Marian D. Rötzer, Claron J. Ridge, Florian F. Schweinberger, Ueli Heiz, Bokwon Yoon, Uzi Landman

**Affiliations:** 1 Chair of Physical Chemistry, Catalysis Research Center, Chemistry Department, Technische Universität München, Lichtenbergstraße 4, Garching 85748, Germany; 2 Air Force Research Laboratory, Energetic Materials Branch, 2306 Perimeter Road, Eglin AFB, Florida 32542, USA; 3 School of Physics, Georgia Institute of Technology, Atlanta, Georgia 30332-0430, USA

## Abstract

The sensitivity, or insensitivity, of catalysed reactions to catalyst structure is a commonly employed fundamental concept. Here we report on the nature of nano-catalysed ethylene hydrogenation, investigated through experiments on size-selected Pt_*n*_ (*n*=8–15) clusters soft-landed on magnesia and first-principles simulations, yielding benchmark information about the validity of structure sensitivity/insensitivity at the bottom of the catalyst size range. Both ethylene-hydrogenation-to-ethane and the parallel hydrogenation–dehydrogenation ethylidyne-producing route are considered, uncovering that at the <1 nm size-scale the reaction exhibits characteristics consistent with structure sensitivity, in contrast to structure insensitivity found for larger particles. The onset of catalysed hydrogenation occurs for Pt_*n*_ (*n*≥10) clusters at *T*>150 K, with maximum room temperature reactivity observed for Pt_13_. Structure insensitivity, inherent for specific cluster sizes, is induced in the more active Pt_13_ by a temperature increase up to 400 K leading to ethylidyne formation. Control of sub-nanometre particle size may be used for tuning catalysed hydrogenation activity and selectivity.

## Abstract (web summary)

Ethylene hydrogenation often serves as an example of a structure insensitive reaction. Here, the authors study the reaction, catalyzed by a range of size-selected platinum nanocatalysts, and demonstrate that in the sub-nanometer regime particle size can be used to tune hydrogenation activity and selectivity.

## Graphical abstract

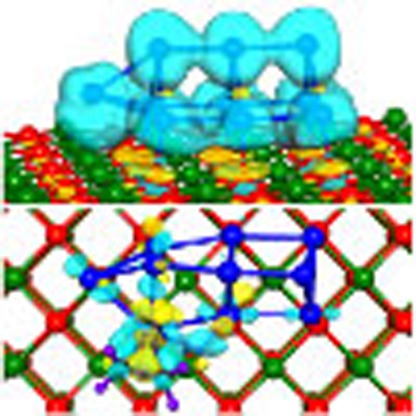

The classification and systematization^1^,^2^,^3^ of heterogeneously catalysed reactions as ‘demanding' or ‘facile', with the former term describing dependence on catalyst particle size and the latter associated with reactions that are size invariant, has been a central guiding theme of research in heterogeneous catalysis for over half a century^4^,^5^,^6^,^7^. The early focus on particle size came from the realization that the fraction of atoms situated at a metal particle's surface to the total number of metal atoms, sometimes termed the ‘fraction of exposed atoms'^6^ or ‘degree of dispersion'^1^, varies with particle size, approaching unity for very small particles (diameter ≤1 nm) and taking small values for large particles. It was recognized early, based on simple geometric (polyhedral) models (tetrahedral, octahedral, cuboctahedral and so on), that the above-mentioned size-related classification may be related to the size-dependent abundance of atomic configurations characterized by surface atoms with low coordination numbers (for example, edges or corners). These under-coordinated atoms were expected to express varying proclivities for engaging in chemical reactions depending on the nature of the reactants and the ambient conditions^1^,^4^,^5^,^6^.

The advent and proliferation of atomic and molecular scale surface science preparation and characterization techniques in the 1970s focused on reactions catalysed on well-defined single-crystal model systems. It then became customary (extending up to date) to classify heterogeneously catalysed reactions as structure sensitive or structure insensitive, corresponding to the aforementioned classifications of demanding and facile, in order to better describe the primary difference between these single-crystal model systems. An example of a structure-sensitive reaction is ammonia synthesis through the reaction of nitrogen and hydrogen on iron single-crystal surfaces where the Fe(111) surface exhibits a higher turnover frequency (TOF) than Fe(100) or Fe(110)^8^. Ethylene hydrogenation catalysed by platinum serves as an example of a structure-insensitive reaction, that is, no change in the TOF has been observed on different single-crystal planes^9^. The latter fundamental and technological important reaction stands out as the first reaction for which a catalytic mechanistic pathway has been formulated (the Horiuti-Polanyi mechanism, HP), and it forms the focus of our investigation in this paper^10^.

The classification of the ethylene hydrogenation as structure insensitive is supported by decades of experimental work employing mostly surface science techniques for reactions taking place on well-characterized extended transition metal surface planes (see Supplementary Note 1 for details), or investigations on samples made of surface-supported distributions of relatively large metal particles with sizes ranging from 1.5 to 10 nm diameter. Although full understanding pertaining to the microscopic origins of the observed structure insensitivity is still lacking^5^,^7^,^11^,^12^, a commonly cited view holds that formation of carbonaceous species (in particular ethylidyne) in the course of the hydrogenation reaction, leads to saturation coverage (poisoning) of the metal surface that results in masking of structural features that could have otherwise brought about different reactivities, and/or alternative reactive outcomes, for surfaces showing different crystallography; that is, if not for the formation of poisonous carbonaceous species, metal surfaces with different crystallography (that is, different Miller indices) would have exhibited structure sensitivity.

Metal particles with sizes reduced to the <1 nm in ‘diameter' range (containing 10–20 atoms) may be viewed as quasi-zero-dimensional (0D) quantum dots, characterized by electronic quantum size effects^13^,^14^ and specific atomic organization motifs, that lead to the emergence of unique size-dependent physical and chemical properties. In this regime, extrapolations from larger sizes using arguments relying on surface-to-volume ratios and scaling relations based on the enumeration of special sites (for example, corner and edge atoms) as a function of the size are not operative^15^,^16^,^17^. In this size scale—that is, in the limit of ultimate dispersion—where almost all of the atoms of the metal cluster are essentially surface atoms and where almost all the cluster atoms can be classified as under-coordinated (compared with the bulk), a new materials' size regime is entered^13^,^14^,^18^,^19^. Explorations of materials' properties in this limiting reduced-size scale requires experiments and first-principles theoretical treatments with atom-by-atom resolution. This is indeed the approach that we have taken in this study.

In quest of appraising the applicability of the fundamental concept of structure sensitivity/insensitivity to nanoscale catalysts, founded on the basis of observations made at larger size scales, we focus here on the hydrogenation of ethylene catalysed by size-selected platinum nanoclusters containing between 7 and 40 Pt atoms supported on an MgO(100)/Mo(100) surface, which is inactive for ethylene hydrogenation. As previously mentioned, ethylene hydrogenation serves often as a generic example of a structure insensitive reaction, obeying the parallel reactions selectivity criterion (PRSC)^1^, with the full-hydrogenation-to-ethane channel and the alternative half-hydrogenation-to-ethyl/dehydrogenation-to-ethylidyne pathway serving as the operative two parallel reactions. The PRSC stipulates that: ‘The best way to proceed is to choose a molecule reacting along two parallel paths and measure the selectivity defined as the rate of the two parallel reactions. If the two products come from different adsorbed states requiring different surface structures, a change of selectivity with dispersion or mode of preparation of the metal may be found. The most unequivocal case is when the specific activity for one of the parallel reactions changes from one catalyst to the next, whereas the specific activity for the other remains unchanged”. Thus, our present experimental and theoretical studies of the ethylene hydrogenation reaction catalysed by nanoscale platinum clusters with atom-by-atom size-controlled resolution, joined with the first adaptation of the PRSC to nanoscale catalysts, provides, as elaborated below, benchmark results pertaining to the reaction's structure sensitivity/insensitivity at the bottom of the catalyst particle size range. As in the case of larger catalyst particles, both the ethylene-hydrogenation-to-ethane channel and the parallel ethylidyne-producing, poisoning, route must be considered, culminating in the fundamental uncovering that at the ultimate <1 nm size scale, the ethylene hydrogenation reaction catalysed by supported platinum particles exhibits characteristics consistent with being a structure-sensitive reaction, in contrast to the accepted structure insensitivity of the reaction found for particles with a lower dispersion.

## Results

### Research plan

We begin with temperature programmed reaction (TPR) experiments, starting from the low temperature adsorption of the two reactants (H_2_ and C_2_H_4_) where all reaction pathways are quenched (except H_2_ dissociation which may occur spontaneously even at low temperatures, see Methods for details). Subsequent measurement of the thermally desorbed species (reaction products and/or unreacted molecules), made at increased temperature, yields an effective hierarchical scan of the spectrum of activation energies operative in the catalysed reaction under study; for technical detail see Methods. It should be noted that the MgO(100) thin film used in this study has been determined to be defect poor^19^,^20^,^21^ by a combined metastable impact electron spectroscopy (MIES) and temperature programmed desorption (TPD) study (see Methods and Supplementary Figs 2–4 for the spectroscopic and chemical characterization of the thin film). Analysis of the results of the above-mentioned characterizations lead us to conclude that the films prepared for the investigations described herein are essentially free of surface defects such as F-centres or other under-coordinated electron trapping sites, and that effects because of roughness, disorder effects, island formation and grain boundaries/corners/steps, are of no consequence (or below the detection limit) for the interpretation of the measurements and analysis presented in this paper.

The information obtained from these measurements is directly amenable to analysis and comparison with *ab initio*, first-principles, theoretical calculations (see Methods for details) that provide microscopic insights about the reaction mechanisms. To go beyond the above one-catalytic-cycle experiments, the reactivity of the size-selected Pt clusters is subsequently studied under isothermal, quasi-steady-state, conditions with the use of a pulsed molecular beam of ethylene. Under such circumstances, all the reaction channels that open at temperatures up to the selected temperature of the experiment are operative, including parallel reaction paths that are potentially detrimental to the full hydrogenation of ethylene to ethane, namely the aforementioned coking reactions such as the one resulting in the formation of ethylidyne.

### One-catalytic-cycle experiments

**Figure 1:**
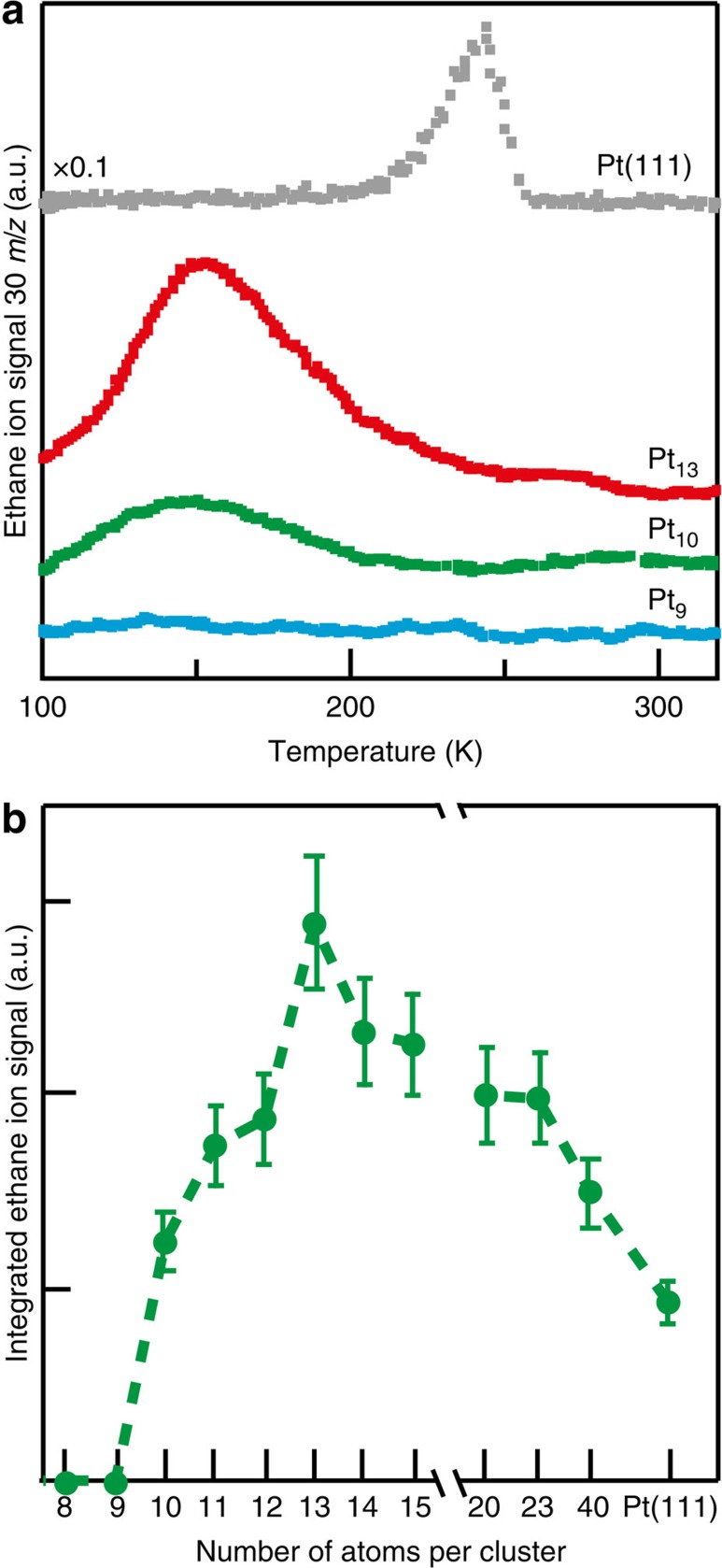
TPR of ethylene hydrogenation on Pt_*n*_ clusters and Pt(111). (**a**) The measured ethane ion signals (*m*/*z*=30) for Pt_9_, Pt_10_, Pt_13_ and Pt(111) as a function of substrate temperature are shown. (**b**) The integrated signal from the curves in **a** as well as for cluster sizes up to Pt_40_ normalized to the number of Pt atoms, are displayed. The TPR experiments were performed by first dosing 0.4 H_2_ molecules per MgO surface atom (1 cm^2^=2.25 × 10^15^ surface atoms) followed by 0.4 C_2_H_4_ ethylene molecules per MgO surface atom at 100 K followed by applying a temperature ramp of 2 K s^−1^. The error bars represent a 16% error, which was determined from multiple measurements on a single cluster size.

Representative cluster-TPR spectra depicted in Fig. 1a and compared with one measured for a Pt(111) surface show negligible reactivity of the Pt_9_ cluster and ethane production for Pt_10_ and Pt_13_ peaking at ∼150 K, that is ∼100 K below the temperature measured for the extended Pt(111) surface. This indicates similar activation energies for clusters in this size range that are considerably lower than the one found for the reaction catalysed on the Pt(111) surface. The integrated areas under the measured TPR curves, normalized to the number of atoms per cluster (Fig. 1b) illustrate that the supported platinum clusters catalyse the formation of more ethane per Pt atom than the corresponding amount generated from the Pt(111) surface, with a maximum found for Pt_13_. Most importantly, the temperature-dependent variation of the ethylene hydrogenation reactivity of the Pt_n_ (*n*=9, 10 and 13) clusters as a function of their size and, in particular, the appearance of a threshold size (*n*=10) for onset of the catalytic activity, suggest that ethylene hydrogenation catalysed by Pt nanoclusters in the 1 nm size range falls under the classification of a size (structure)-sensitive reaction, in contrast to the commonly accepted structure insensitivity when catalysed by larger particles and extended surfaces. As demonstrated below, this conjectured new classification in the sub-nanometre size range is indeed supported by measurement of TOFs manifesting size (structure) sensitivity under steady-state conditions at room temperature (300 K).

### Structures and reaction mechanisms from *ab-initio* calculations

In light of the above-observed size dependencies, it is evident that uncovering the atomic-arrangement motifs of the sub-nanometre-size supported Pt cluster catalysts is imperative to advancing our understanding of the physical principles underlying the above-noted apparent structural sensitivity of the catalysed reactions. The development and implementation of efficient and practical optimization strategies (global optimization in particular^22^) is a vexing problem of great importance in diverse fields, including the physical sciences, where we mention continuing efforts in the areas of protein folding, and the atomic arrangements in biomolecules, clusters and crystals^23^. A main difficulty in this area originates from the fact that the global extremum of a real multivariate function (for example, the total potential energy of an atomic cluster as a function of the locations of the atoms) is actually a local property, thus requiring an exhaustive search. Consequently, a large number of diverse methods for global structure optimizations have been proposed (see, for example, refs 22, 24 and citations therein). Although significant progress has been achieved, proving that the global extremum has indeed been found seems to be a rather unattainable task for most systems of interest^22^.

**Figure 2:**
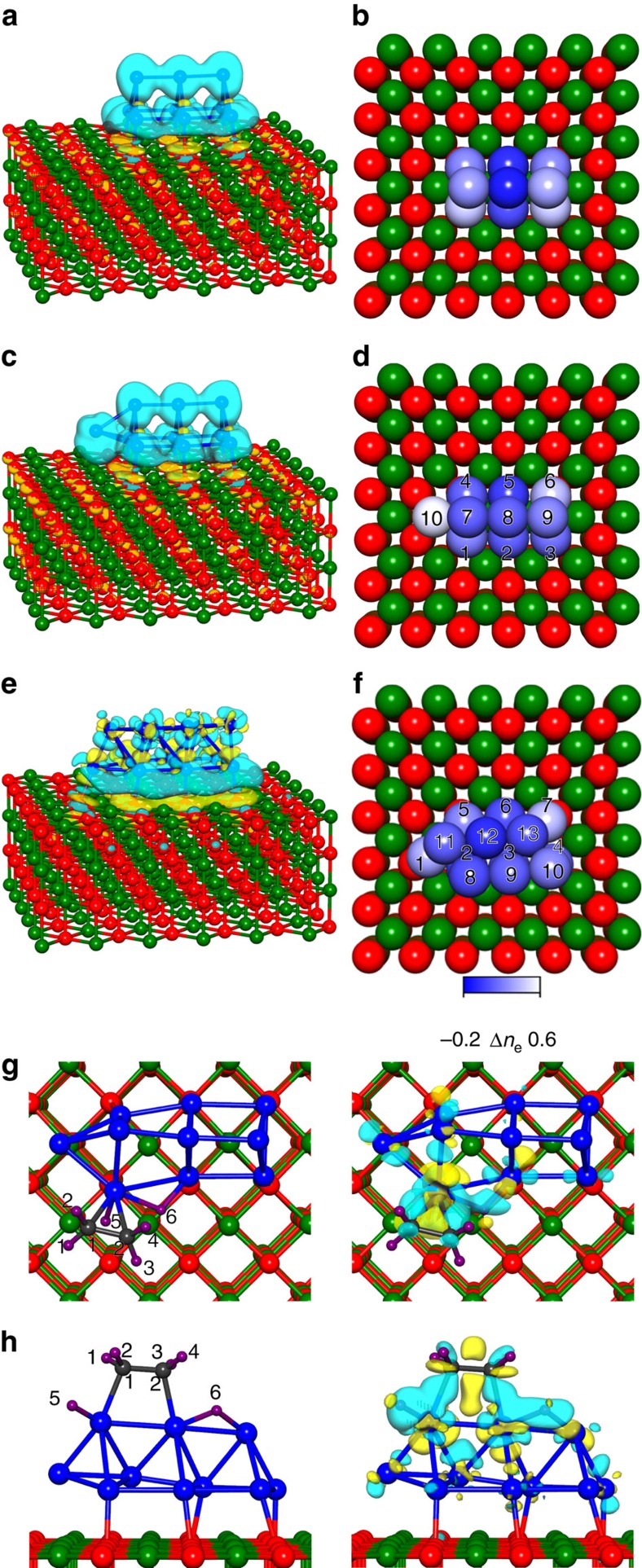
Optimal configurations and charge distribution of bare Pt_n_/MgO and co-adsorbed C_2_H_4_+H_2_. **a**, **c** and **e**, corresponding to Pt_9_, Pt_10_ and Pt_13_ clusters, the blue and yellow contour hyper-surfaces correspond to excess (light blue) and deficient (yellow) charge distributions obtained as the difference between the total charges before and after adsorption of the clusters; these hypersurfaces are drawn such that the excess electronic (negative) charge inside the light blue hypersurface is 50% of the total electronic charge and the same for the positive charge inside the yellow hypersurfaces. Bader charge analysis is given in **b**,**d** and **f**, with lighter colour corresponding to excess number of electrons (that is, excess negative charge on the corresponding atom); for the values of the Bader charges, see Supplementary Figs 14 and 15. Co-adsorption of C_2_H_4_ and H_2_ on Pt_10_/MgO is shown for the π (**g**) and di-σ (**h**) bonding modes. The adsorption geometries are shown on the left, and on the right, we depict the bonding frontier orbitals of the adsorption system (the light blue and yellow denote different signs of the wave function); the σ-type Pt-C bonds are clearly seen in **h** (note the directed wave function contours on the right). In both **g** and **h**, atoms 5 and 6 are the proximal dissociated co-adsorbed H atoms.

The optimal (lowest-energy) structures of the surface-supported Pt_9_, Pt_10_ and Pt_13_ clusters determined through first-principles calculations (based on extensive searches, guided by cluster structural motifs gathered for other systems and by previously proposed search methodologies^24^) are displayed in Fig. 2 as well as in Supplementary Figs 7–15. It is evident that these clusters prefer a three-dimensional (3D) prismatic two-layer structure. In addition, we observe that these adsorbed clusters possess a large number of higher-energy isomeric structures (with the preferred ones having 3D geometries), reflecting the complex nature of their potential energy surface; see in particular the extensive rich spectra of structural isomers in Supplementary Figs 7–13. Although we focus in the following on the lowest-energy clusters, it is possible that some higher-energy isomers are also present on the supporting surface.

It is pertinent to remark here that the optimal (minimum total energy) configurations, as well as those of the higher-energy isomers, were determined through unconstrained relaxation of the atomic positions in conjunction with density functional calculations of the total energy (see Methods for details). The bare clusters were positioned on the MgO(100) surface, and their configurations, together with the positions of the atoms of the underlying Mg(100) substrate, were optimized via unconstrained relaxations. Once the optimal cluster structures have been determined, the binding (adsorption) energies and atomic positions of the reactants (that is, the individual H_2_ and C_2_H_4_ molecules, as well as multiply adsorbed, and co-adsorbed, systems) were determined through total energy minimization; similarly, in determination of the reaction path, the degrees of freedom of all atoms of the system (substrate, cluster and reactants) have been relaxed except for the very few describing the chosen reaction coordinate (for example, the distance, or combination of distances, between atoms of the reactants), which is varied in a stepwise manner (recording the total energy change along the change in the reaction coordinate yields the reaction energy profile and the activation barrier for the reaction (see Methods for details)).

The relaxation procedure described above allows for adsorbate/reactant-induced structural relaxations of the underlying metal cluster and metal-oxide (MgO(100)) substrate; this form of adsorbate-induced cluster-catalyst relaxation (Supplementary Fig. 16 where inter-isomer structural crossover is shown) is a manifestation of a more general class of cluster relaxation processes termed fluxionality^14^,^18^, which includes also cluster relaxation in the course of a chemical reaction, named dynamical reaction-induced cluster fluxionality^18^. By considering systematically only relaxed configurations, the size-dependent fluxional propensity of clusters in the size regime studied here becomes one of the characteristics that determine the response of the clusters to the presence of reactants and intermediates (for example, ethylidyne) and the consequent size-dependent reactivities and the course of reactions catalysed by these clusters. Finally, in addition to being of physical significance in the description of the mechanisms of cluster-catalysed reactions, the inter-isomer structural conversion shown in Supplementary Fig. 16 illustrates the use of the dynamical reaction-induced cluster fluxionality as a method for uncovering structural isomers; in fact, it has been used by us to discover the ground-state structure of the metal cluster in Pt_13_/MgO (see Fig. 2e,f and Supplementary Figs 11 and 16).

The spin state of the bare-adsorbed Pt clusters is *N*_↑_-*N*_↓_=2 (where *N*_s_ is the number of electrons with spin s=↑ or ↓), and they are found, through total charge differences (calculated before and after adsorption and shown in Fig. 2a,c,e and Bader charge analysis (BCA, see Fig. 2b,d,f and Supplementary Figs 14 and 15)), to be negatively charged with the total excess Bader electronic charges on the adsorbed cluster being: *δρ*_B_[Pt_*n*_/MgO]=1.68 e, 1.78 e and 1.92 e for *n*=9, 10 and 13, respectively. We find that whereas the total excess electronic charges on the *n*=9 and 10 clusters are quite similar, close to a third (0.51e) of *δρ*_B_[Pt_10_/MgO] is located for the Pt_10_ cluster on the capping atom (the 10th atom in Fig. 2d); similarly, a large contribution to *δρ*_B_[Pt_13_/MgO] is also found on the capping atom (the first atom (marked 1) of Pt_13_, shown in Fig. 2f, which is found to have an excess local charge of 0.34 e out of a total of 1.92 e, with the atoms marked 7 having also a large excess charge of 0.36 (Supplementary Fig. 15). For the Pt_10_ cluster, this excess charge distribution leaves a smaller amount of excess charge (1.78 e −0.51 e=1.27 e) to be distributed over the remaining nine atoms of the Pt_10_ cluster in comparison with the total excess charge (1.68 e) available for distribution over the compact (no capping atom) Pt_9_ triangular prism cluster. This smaller amount of negative excess charge on most of the atoms of the Pt_10_ cluster compared with the Pt_9_ case, are found to influence binding sites, adsorption energies and consequent reaction pathways on the adsorbed clusters as well as demonstrating that, in this size-range, even the addition of a single atom, while not changing the basic geometric structure, can drastically alter the electronic properties of a cluster.

Previous investigations (see review in ref. 7) identified two adsorption modes of ethylene on the (111) surface of platinum, the π and di-σ modes, where in the former the ethylene is thought to coordinate to a single Pt atom through a π bond, with the ethylene maintaining (at least to a large extent) sp^2^ hybridization, and in the latter the adsorbed molecule is attached to two adjacent Pt atoms (in a η^2^ manner) through two σ bonds showing a significant extent of sp^3^ hybridization. On the extended Pt(111) surface, the π-bonded ethylene was found to be the preferred adsorption configuration only at very low temperatures, whereas for *T*=100–240 K di-σ ethylene is preferred under ultra-high vacuum (UHV) conditions, with ethylidyne forming at higher temperatures. All the theoretical computational studies^25^ corroborated the experimentally observed preference for di-σ over the π ethylene absorption mode, whereas the weakly adsorbed π-bonded species is experimentally believed to be the main reactant during hydrogenation of ethylene^9^.

**Figure 3:**
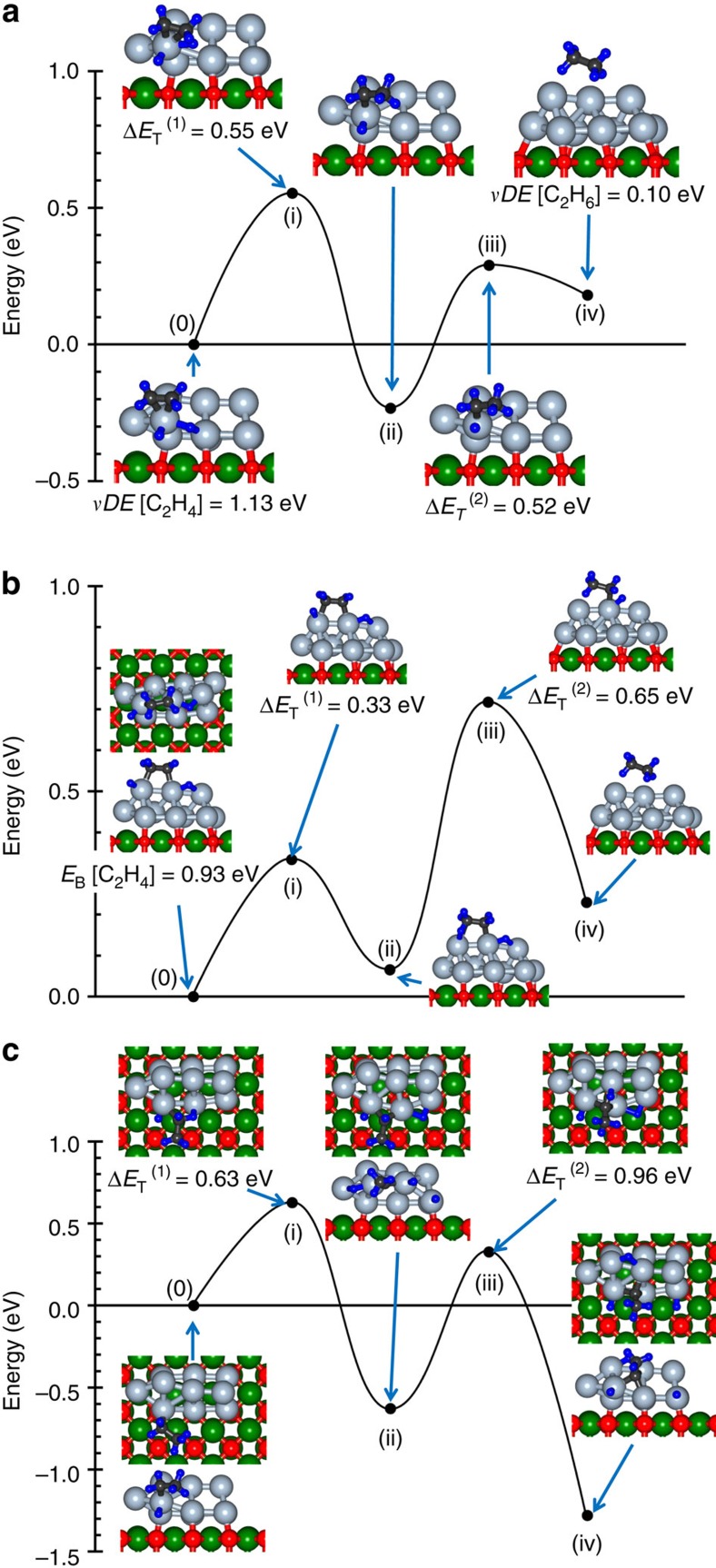
Calculated first-principles steered reaction pathways catalysed by Pt_10_/MgO. (**a**,**b**) Low-activation-barrier reaction pathways for the π (**a**) and di-σ (**b**) bonded ethylene (configuration on the left, marked 0), co-adsorbed with dissociated hydrogen. The activation energy barriers are denoted as Δ*E*_T_^(*k*)^, *k*=1,2. The reaction proceeds through the two successive hydrogenation steps described in the Horiuti-Polanyi mechanism. (**c**) SRP for the low-temperature generation of ethylidyne (≡CCH_3_) on Pt_10_/MgO, starting from the ethyl (-CH_2_CH_3_) intermediate (configuration (0), on the left) generated in the first step of the reaction for the π-bonded ethylene (depicted as configuration (ii) in **a**). The two activation barriers correspond to dehydrogenation processes, resulting in a strongly adsorbed ethylidyne molecule (≡CCH_3_, configuration (iv)). Surmounting the barrier for the second dehydrogenation process is assisted by the highly exothermic (∼1.35 eV) formation of the -CHCH_3_ intermediate (iii) resulting from the first dehydrogenation step.

**Figure 4:**
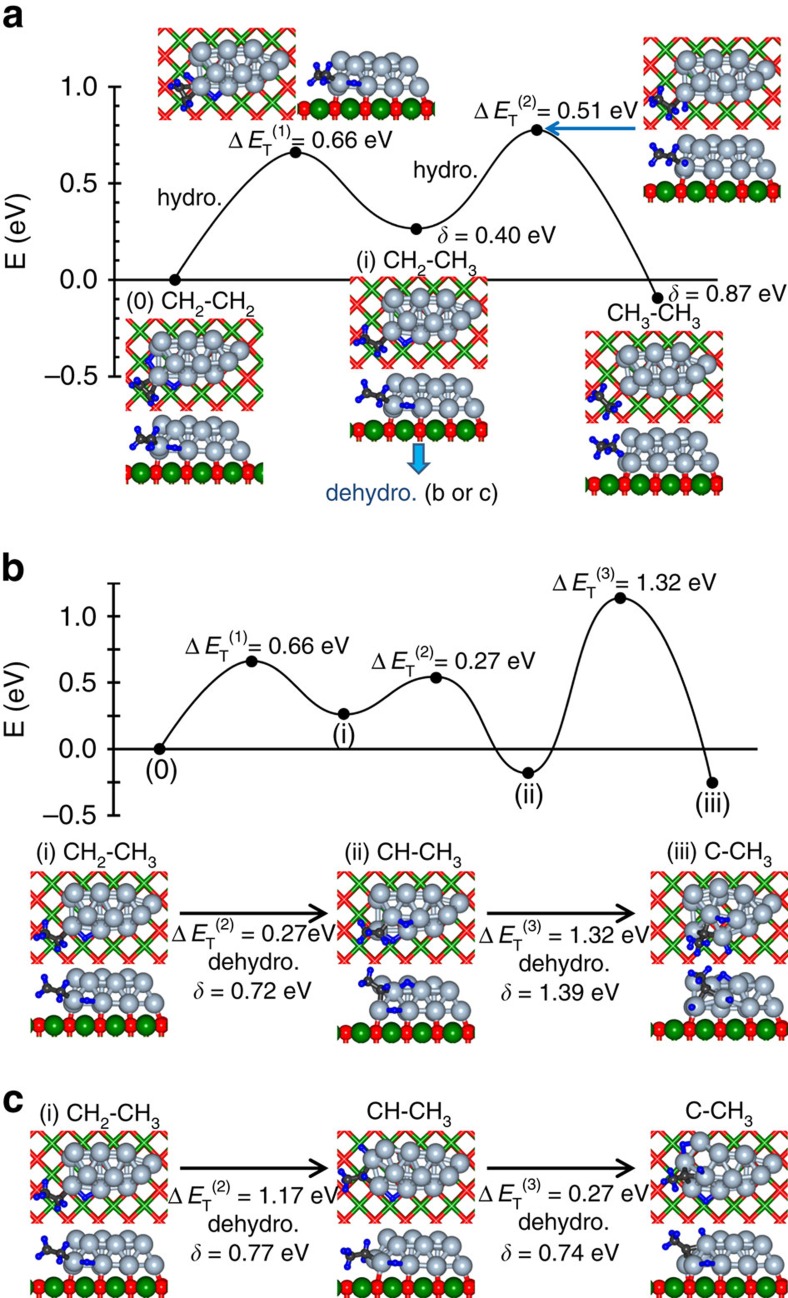
Calculated first-principles steered reaction pathways catalysed by Pt_13_/MgO. (**a**) Low-activation-barrier reaction pathway for hydrogenation of π-bonded ethylene configuration co-adsorbed with dissociated hydrogen on Pt_13_/MgO. The reaction proceeds through the two successive hydrogenation steps described in the HP mechanism. The blue downward arrow near configuration (i) indicated the adsorbed ethyl intermediate (i) is the starting configuration for the **b** and **c** pathways. (**b**,**c**) Alternative reaction channels yielding ethylidyne (≡C-CH_3_), starting from the π-bonded ethylene configuration co-adsorbed with dissociated hydrogen (marked (0) in **a**). The ethylene hydrogenation reaction producing ethane (see pathways in **a**) entails step (0)→(i), with Δ*E*_T_^(1)^=0.66 eV, followed by the activated second hydrogenation (Δ*E*_T_^(2)^=0.51 eV) that yields a weakly adsorbed C_2_H_6_ molecule. Two alternative dehydrogenation reaction channels that lead to formation of ethylidyne (starting from the ethyl, CH_2_CH_3_, intermediate, marked (i) in **a**, see blue downward arrow in **a**) are shown in **b** and **c**. The **b** and **c** pathways entail higher energy barriers (1.32 eV and 1.17 eV, in **b** and **c**, respectively) and thus their activation requires heating above room temperature, resulting in marked poisoning of the ethane-producing hydrogenation channel (see Fig. 5). The graph (profile) of the reaction path is shown only for channel **b**. The energy difference between the top of an activation barrier and the subsequent local minimum on the reaction pathway is given (in eV) by δ; this energy gives the height of the reverse reaction step, or it can assist (in part) passage over the subsequent energy barrier.

Contrary to extended surfaces, for the magnesia-supported Pt_*n*_ (*n*≥9) clusters, we find essentially the same propensity for adsorption in the π or di-σ mode, with the corresponding vertical desorption energies (VDEs) of the C_2_H_4_ molecules (co-adsorbed with a dissociated H_2_ molecule in neighbouring sites) calculated to be VDE=1.13 eV and 0.93 eV for the π and di-σ modes, respectively (see Figs 2g,h and 3a,b for details); note, in particular, the much higher value of the desorption energy for the π-bonded molecule compared with the corresponding calculated (0.87 eV (ref. 25) and measured (0.41±0.10 eV (ref. 26) values on the Pt(111) surface. Furthermore, the π-bonded molecule (see Figs 2g and 3a for adsorption on Pt_10_/MgO)) has a C–C bond length *d*(C–C)=1.425 Å, which is close to the experimental value of 1.41 Å (ref. 27) this value is somewhat larger than *d*(C–C)=1.334 Å value found in the isolated C_2_H_4_ molecule, indicating a certain degree of sp^3^ rehybridization. In the di-σ adsorbed ethylene molecule, we find evidence for a larger degree of sp^3^ hybridization, with *d*(C–C)=1.469 Å, which is closer to the value found in the isolated C_2_H_6_ molecule (*d*(C–C)=1.528 Å). Similar results were found by us for the two adsorption modes of ethylene on the supported Pt_13_ cluster (see the π-bonded adsorption configuration, marked 0, with VDE=1.54 eV in Fig. 4a). The charge redistributions for both ethylene adsorption modes reflect the frontier orbital interactions leading to ethylene chemisorption, described by the Dewar–Chatt–Duncanson model^28^,^29^ (see also Supplementary Note 2), involving electron donation from the highest occupied (π) ethylene orbital into an empty d orbital of the metal, and back-donation from the filled Pt d-orbitals into the ethylene lowest unoccupied (antibonding) π* orbital. The resulting electron charge isosurfaces (see right panels in Fig. 2g,h) portray the resulting redistribution of the electron density. Both the above bonding processes as well as the repulsive interactions between the filled frontier orbitals of ethylene and occupied d-states of the metal catalyst particle (termed as Pauli^30^,^31^ or four-electron repulsion^32^) depend on: (i) coordination of atoms making up the Pt particle, with reduced coordination, such as at edge and corner atoms, resulting in decreased repulsion, and (ii) occupation of metal d-states (with the Pauli repulsive interaction between filled orbitals of the metal cluster and the adsorbate increasing at cluster sites having larger excess electron charge); site-coordination and charging patterns can be ascertained from the calculated information given in Fig. 2a–f, and similar considerations apply also to the catalysed ethylene hydrogenation mechanism discussed below. In this context, it is pertinent to recall here our systematic study^33^ of the effect of charging by cluster–substrate interactions on the adsorption and low-temperature combustion reaction of carbon monoxide and dioxygen, catalysed by MgO-supported gold clusters of sizes similar to those considered in the current investigation.

To gain insights into the microscopic ethylene hydrogenation mechanism, we carried out extensive first-principles steered reaction-pathway (SRP) simulations. For Pt_9_, no low-activation-energy hydrogenation channel was found in the simulations (see Supplementary Figs 17–20 for details), in agreement with the TPR results (Fig. 1). On the other hand, multiple reaction pathways, characterized by relatively low activation barriers, were found in SRP simulations for the larger clusters, as illustrated in Figs 3 and 4 for Pt_10_ and Pt_13_, respectively.

The simulated pathways starting from a π-bonded ethylene molecule co-adsorbed with a dissociated H_2_ on the Pt_10_ and Pt_13_ clusters are shown in Figs 3a and 4a, respectively. For both cluster sizes, we observe two successive energy barriers (denoted as Δ*E*_T_(*i*), *i*=1,2) corresponding to the two hydrogenation steps of the HP mechanism, whose heights are sufficiently low to permit the experimentally observed low temperature reactivity, see Fig. 1 for details. In the first reaction step (marked (0)-(i) in Fig. 3a for Pt_10_/MgO), one of the adsorbed hydrogen atoms approaches the nearest carbon atom (initially *d*(C^(1)^–H^(1)^)=2.432 Å). The first activation barrier Δ*E*_T_^(1)^=0.55 eV, is characterized by *d*(C^(1)^–H^(1)^)=1.25 Å and *d*(C^(1)^–C^(2)^; ΔE_T_^(1)^)=1.487 Å (compared with *d*(C–C)=1.487 Å calculated for gaseous ethyl C_2_H_5_) portraying formation of an adsorbed ethyl-like top-of-the-barrier intermediate. In the local minimum (marked (ii)) that follows the first barrier *d*(C^(1)^–H^(1)^)=1.105 Å, *d*(C^(1)^–C^(2)^; (ii))=1.529 Å, and the adsorbed ethyl intermediate is inclined with respect to the cluster (*d*(C^(1)^–Pt^(1))^=3.065 Å and *d*(C^(2)^–Pt^(1))^=2.080 Å). The closer value at the barrier-top of *d*(C^(1)^–C^(2)^; Δ*E*_T_^(1)^) to *d*(C^(1)^–C^(2)^; (ii)) then to the initial state of the adsorbed ethylene, *d*(C^(1)^–C^(2)^;(0))=1.425 Å, qualifies Δ*E*_T_^(1)^ as a ‘late transition state barrier'. Full hydrogenation occurs in the second activation process—(ii) C_2_H_5_→(iii) Δ*E*_T_^(2)^=0.52 eV→(iv) adsorbed C_2_H_6_ with VDE=0.1 eV—resulting in the product ethane molecule. The pathways of the hydrogenation reaction of the di-σ-adsorbed ethylene molecule (shown for Pt_10_/MgO in Fig. 3b) resemble the one described above, with the first activation barrier being lower (0.33 eV); for geometric details of the reaction pathways, see Supplementary Figs 21–27. Similar results were found in our SRP simulations for the Pt_13_/MgO cluster (see Fig. 4a for the π-bonded ethylene molecule); for details, see Supplementary Figs 28–36).

The principal result of our analysis to this point is the discovery and explanation of the onset of low-temperature ethylene hydrogenation processes (peaking at temperatures of ∼150 K) on magnesia-supported 3D Pt_*n*_ clusters for *n*≥10, with the calculated activation barriers for *both* the π and di-σ C_2_H_4_ adsorption modes on the Pt cluster catalysts being significantly smaller than those obtained for the Pt(111) surface; compared with values calculated for the lowest coverage (1/9) on Pt(111)^25^. This reflects significant enhancement of the hydrogenation reaction, achieved through the use of supported platinum clusters of <1 nm diameter (on the order of 10–15 Pt atoms); note, however, the absence of catalytic activity of 3D Pt_*n*_ clusters with *n*≤9. The measured larger hydrogenation reactivity of the Pt_13_ cluster correlates with the calculated larger number of active sites on this cluster for both H_2_ dissociative adsorption (see Supplementary Fig. 1 and Methods for details), and low-barrier hydrogenation of co-adsorbed ethylene and hydrogen.

In addition to the low-activation-energy pathways found on the Pt_*n*_/MgO (*n*≥10), we have found on these clusters (particularly on the larger cluster, Pt_13_/MgO) several reaction sites characterized by higher ethylene-hydrogenation activation energy barriers; typically, Δ*E*_T_>0.8–1.0 eV (see Supplementary Figs 21–36 for details). Most significantly, along with the low-temperature hydrogenation pathways (Fig. 3a,b) we found for the Pt_10_/MgO catalyst a relativelyc low-barrier dehydrogenation reaction channel resulting in the formation of ethylidyne (Fig. 3c), whereas for the larger Pt_13_/MgO cluster we found multiple high-energy ethylidyne formation pathways; see Fig. 4c,d manifesting energy-barriers of Δ*E*_T_∼1.2–1.3 eV. This finding implies for Pt_13_ an onset of significant ethylidyne formation upon heating to higher temperatures (*T*∼350–400 K), which acts as a blocking, or poisonous, agent for ethylene hydrogenation; for the Pt_10_ clusters such alternative reaction channels are open already at 300 K, see, for example, Fig. 3c.

### Isothermal multi-catalytic-cycle experiments

The pulsed molecular beam technique was applied to determine TOFs under quasi-steady-state isothermal conditions (see Methods for experimental details). This method allows probing and verification, under actual catalytic conditions, of the trends deduced from the TPR experiments, as well as confirmation of the predictions obtained from first-principles calculations regarding ethylidyne formation (see above). At these elevated temperatures, processes such as cluster sintering become more probable, but our data did not show any indication that this was occurring during the time scale of our experiments and studies performed on size-selected clusters also cast strong doubt that sintering occurs already at 400 K, see Methods for further discussion.

**Figure 5:**
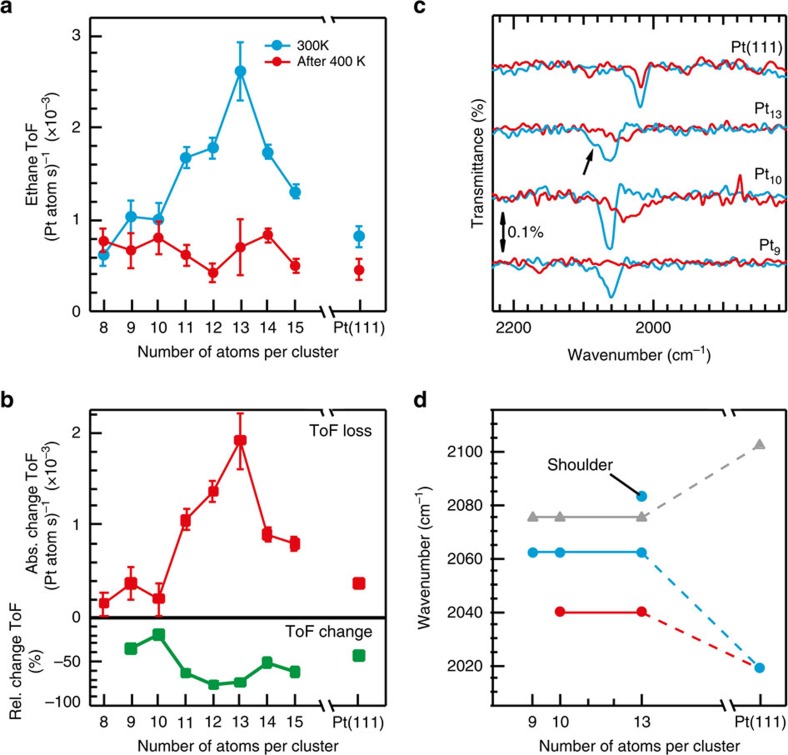
Pulsed molecular beam of C_2_H_4_+H_2_ reacting on Pt_*n*_/MgO along with infrared spectra. (**a**) Measured TOF at 300 K of the ethylene hydrogenation on Pt_8–15_ clusters and Pt(111) single-crystal surface, before (blue) and after (red) running the identical reaction at 400 K. The measured TOF has been normalized to the number of platinum atoms for Pt_8–15_ and the Pt(111) surface. A background deuterium pressure of 2 × 10^−6^ mbar was used for all experiments. (**b**) Decrease of the turnover frequency between the blue and red measurements as well as this value represented as a percent loss. (**c**) Infrared reflection absorption spectra of 10 L CO after the experiments in **a** measured at 100 K. Blue spectra were taken after pulsing at 300 K and red after the clusters had been exposed to the same reaction conditions at 400 K and again at 300 K. (**d**) Position of the CO stretch on clean cluster samples (grey data points) as well as after the blue and red data points were acquired. The error bars represent the standard deviation of multiple activity measurements on the same cluster size.

The measured ethane TOFs at 300 K displayed in Fig. 5a (blue) show that at this temperature Pt_*n*_ clusters with *n*<10 are active, as expected from the calculated activation energies on Pt_9_. Furthermore, at this temperature, the distinct onset of reactivity for n>9 observed in the TPR, one-catalytic-cycle, experiments (see Fig. 1 for details) is not operative, and reactivity variations are observed only in a narrow size window for Pt_11_–Pt_14_, with a pronounced maximum activity for Pt_13_. This demonstrates the emergence of a size window where the reaction is *size* (structure) sensitive, in contrast with the size (structure) insensitivity observed for other cluster sizes, nanoparticles and single-crystal surfaces^9^,^34^,^35^,^36^. This serves to illustrate that clusters (of the same chemical identity but different sizes) can exhibit both structure sensitivity and insensitivity and that a single atom can change this designation.

To investigate the influence on the hydrogenation of ethylene brought about by surface species generated by alternative reactions, for example, the aforementioned ethylidyne production channel, we have repeated the measurement of the TOF at 300 K, but this time after the clusters were exposed to 10 pulses of ethylene at 400 K in the presence of deuterium (Fig. 5a, red curve). The absolute change of the TOF for the sizes measured in Fig. 5a, depicted in Fig. 5b, clearly shows that Pt_13_, as well as the other clusters within the enhanced reactivity size window shown in Fig. 5a, deactivate as a result of the 400 K heating step, whereas the other sizes show no significant change in activity. After the 400 K step, all cluster sizes show TOFs similar to the one measured on Pt(111), that is, the reaction has turned size insensitive. This observation is consistent with the results of the calculations (Figs 3c and 4c,d), where the activation barrier for the dehydrogenation pathway to the (hydrogenation blocking) ethylidyne species was found to be thermally accessible already at temperatures as low as *T*<300 K for Pt_*n*_ (*n*≤10), whereas for Pt_13_ ethylidyne production was predicted to require heating to *T*>350 K. Consequently, the size-insensitive clusters, as well as Pt(111), are all passivated already at 300 K, and only with a temperature increase do the more active sizes follow. To optimize reactivity for the hydrogenation of ethylene, it is thus not only essential to choose the right size window between Pt_11_ and Pt_14_, but also the right temperature window 160 K≤*T*≤350 K, where the hydrogenation of ethylene is thermally accessible but not the formation of poisoning species. This size-dependent behaviour of two competing reaction channels offers the potential for controlling and tuning more applied hydrogenation and dehydrogenation reactions on an atom-by-atom basis.

Further evidence for the formation of inhibiting carbon species has been obtained through the use of CO adsorption and infrared reflection absorption spectroscopy to probe the cluster catalysts before and after the reaction, as co-adsorption of carbonaceous species on platinum is known to induce a redshift in the CO stretch frequency because of electron donation to the metal^37^,^38^,^39^,^40^. The attribution of the redshifts reported here to carbon was strengthened by the fact that after the TPR experiment in Fig. 1a, no deuterium adsorption was observed on Pt_9_, Pt_10_ and Pt_13_ indicating a blocking effect of an adsorbate on the clusters. The only plausible explanation would be a dehydrogenated product from ethylene. Although a change in cluster shape could also possibly induce such a small redshift, the inability of this hypothesis to reconcile the TPD and infrared data led to the assignment of co-adsorbed carbonaceous species as the origin of the redshift.

After running the reaction under conditions of highest activity (300 K), ten Langmuir of CO were adsorbed at 100 K and an infrared spectrum recorded (Fig. 5c, blue curves). The CO stretch is red-shifted by 13 cm^−1^ compared with spectra measured from pure metallic sites on all cluster sizes (see the shifted spectra in Fig. 5c and Fig. 5d for both the shifted and unshifted values, respectively). Importantly, the cluster of highest reactivity, Pt_13_, also exhibits a shoulder (marked with an arrow in Fig. 5c) where the CO stretch on a clean cluster is found. In comparison, the Pt(111) crystal shows a much larger redshift (83 cm^−1^, compare the frequencies given by the triangle and filled blue dot on the right in Fig. 5d), which is attributed to adsorbed ethylidyne^26^,^41^,^42^. Performing the same experiment after the 400 K heating steps brings about an even larger redshift of the CO stretch (34 cm^−1^, compare the red and blue curves in the spectra displayed in Fig. 5c, and summarized in Fig. 5d), indicating a larger influence of inhibiting dehydrogenation products as well as complete site blocking on Pt_9_. On Pt(111), the absorption peak decreases slightly in intensity but remains at the same position, which correlates to the expected stability of ethylidyne up to 400 K (ref. 43).

The finding that a CO stretch peak position comparable to that measured for CO on a clean (bare) cluster is found after reaction at 300 K only for the most active size, Pt_13_, reflects the intrinsic resistance of ethylene on Pt_13_ to follow the dehydrogenation pathways leading to ethylidyne formation, as predicted by our theoretical results. The formation of more carbonaceous species on Pt_13_ (portrayed by a larger redshift after pulsing at 400 K) induces the subsequent structure insensitivity, previously established for other cluster sizes, nanoparticles and single crystals.

This combined pulsed molecular beam and infrared reflection absorption spectroscopy study demonstrates for the first time that the formation of carbon species is indeed the key factor underlying structure insensitivity of ethylene hydrogenation on larger particles and extended surfaces of platinum.

## Discussion

The investigations discussed in this paper demonstrate that classification of a reaction as structure sensitive, or insensitive, must be reassessed for materials with sizes in the non-scalable sub-nanometre size regime. This size range has been shown to exhibit surface chemical properties, which can be modulated by a single atom, and where each particle size displays its own mode of behaviour. The previously reported structure insensitivity of ethylene hydrogenation on Pt is shown here to be untenable in the cluster size range of Pt_7_–Pt_40_, where we find structure sensitivity of ethylene hydrogenation to emerge, with maximum reactivity for Pt_13_. Structure-insensitive behaviour was observed to be inherent for specific cluster sizes at ambient temperatures and can be induced in the more active sizes (for example, Pt_13_) by an increase in reaction temperature, which opens dehydrogenation reaction channels leading to the formation of carbonaceous species evidenced in the infrared spectra and predicted by first-principles calculations. Aside from addressing the applicability, at this size range, of common catalyst classification as structure sensitive or insensitive, our findings point to the possibility of controlling the activity and selectivity of hydrogenation and dehydrogenation reactions catalysed by clusters of these sizes. We remark that rather than treating an industrial hydrogenation catalyst under industrial conditions, we focus here, with the use of model catalysts of nanoscale dimensions, on a basic science question of great interest in the field of heterogeneous catalysis pertaining to the concept of structure sensitivity/insensitivity at the bottom of the catalyst size scale. For the catalyst cluster sizes considered here, the reaction is investigated in a temperature range lying mostly below, and up to, room temperature (with certain thermal treatment involving temporal heating to 400 K), which is lower than the temperatures used in typical industrial catalytic hydrogenation applications; the finding that nanocluster catalysts (particularly made of noble metals) catalyse reactions at temperatures significantly lower than larger clusters and extended surfaces made of these metals is rather common now (see, for example, refs 13, 14, 19, 20, 44, 45). Correspondingly, the temperature-window selectivity and thermal-tuning that we discuss and demonstrate are operative, and are kinetically relevant, for these nanocatalysts under the above-specified temperature conditions. This also suggests that in conjunction with future employment of nanocatalysts in industrial-type processes, the concept of temperature selectivity/tuning windows, discussed in this work from a basic science perspective, may emerge also as one of practical relevance.

In the non-scalable size regime, the above findings make element-specific generalizations of catalytic properties, based on single crystals and nanoparticles, rather questionable. The first-principles simulations show that even similar atomic geometric arrangements can lead to widely differing physical and chemical properties. Consequently, each reaction must be individually tested in an atom-by-atom, size-selective, manner, in order to disentangle and reveal the catalytic properties of such systems. The use of structure (in)sensitivity has been a most useful concept for the description and systematization of catalytic processes on single crystals and particles possessing well-defined single-crystal facets. However, with decreasing particle sizes (that is, for higher dispersion), and in particular in the sub-nanometre size regime, extrapolations from systems of larger size are likely to become inadequate, requiring instead an atom-by-atom reassessment.

In closing, the results that we reported here demonstrate that hydrogenation and dehydrogenation reactions can be controlled and tuned by controlling the precise atomic size of the catalyst particle. This has implications for a broad range of chemical processes^46^ ranging from chemical synthesis, to food chemistry, and energy applications, where the insight garnered here, as well as future investigations along these lines, could facilitate a deeper understanding of applied reaction systems, and may offer design strategies for catalysts with enhanced activity and/or selectivity.

## Methods

### Sample preparation and characterization

All experiments were perform in an ultra-high vacuum (UHV) chamber with a base pressure of 1 × 10^−10^ mbar^47^. The Mo(100) (MaTeck, Germany, 0.785 cm^2^) single crystal was cleaned by heating to 2,000 K and subsequent oxidation at 900 K in a 5 × 10^−7^ mbar O_2_ background (5.5 purity, Air Liquide Germany). The purity of the crystal was checked with Auger electron spectroscopy (AES) and ultraviolet photoelectron spectroscopy (UPS)^48^. The MgO(100) film was then grown at 600 K on the Mo(100) single crystal by evaporating a magnesium (≥99.95%, Merck Germany) ribbon in front of the crystal in a 5 × 10^−7^ mbar background of O_2_. After annealing the film at 800 K for *t*>10 min, the film was characterized with AES and is estimated to have a thickness of ten atomic layers, the purity was further confirmed with AES, UPS and MIES.

The presence of surface defects has also been investigated and the films have been determined to be defect poor and exhibit a high degree of order. Oxygen vacancies (F-centres) have been shown to be detectable via MIES as a clear band beginning at a binding energy (BE) of 1 eV and peaking at 2 eV (refs 20, 49). The MIES spectrum in Supplementary Fig. 2 shows this region from the MgO film prepared as described above. No characteristic emission peak below 4 eV is observed, clearly showing the absence of F-centre type defects or other under-coordinated electron-trapping sites.

Surface roughness can also play a factor in thin films and can also influence catalytic activity. Previous surface characterization of thin MgO films concluded that above 7 ML thin film thickness, global surface roughness decreases and (1 × 1) low energy electron diffraction (LEED) spots are visible^50^. As the films grown here are thicker than 7 ML and we have previously published this same LEED pattern from our film growth procedure^21^, the films grown for this study possess low surface roughness.

This same property has also been characterized with the use of MIES, where a narrowing of the O2p band indicates surface ordering^51^. Their spectrum showed a full-width half-maximum O2p peak of 3.2 eV for a disordered film and 2.5 eV for an ordered film. The MIES spectrum in Supplementary Fig. 3 (complete spectrum of Supplementary Fig. 2) shows the full-width half-maximum of the O2p peak (BE=5.5 eV) to be 2.13 eV for the films grown for this study. Again, this is further evidence that the MgO film is highly ordered.

Grain boundaries and islands can be directly detected using a CO TPD. The CO TPD from the thin film used in this study is displayed in Supplementary Fig. 4. The sample was dosed at 100K with 1 CO molecule per MgO surface atom, and a temperature ramp of 2 K/s was applied (see below for technical details regarding the TPD experiment). The spectrum shows only a single, narrow desorption peak at 105 K, which is concordant with a defect poor film. The absence of island formation and the absence of exposed Mo domains in our films was confirmed by the nonappearance of a characteristic desorption peak at about 290 K in our CO TPD measurement (see ref. 52).

In conclusion, the above data clearly show that our films are defect poor, in that they do not exhibit the established spectral and chemical properties expected from F-centres (or other under-coordinated electron trapping sites), rough/disordered films, island-like films and grain boundaries/corners/steps and so on.

The Pt(111) (MaTeck) single crystal was cleaned by cycles of argon ion sputtering at 900 K, followed by oxidation for 5 min at 650 K in a 5 × 10^−7^ O_2_ background pressure and annealing at 1,300 K for 1 min. The purity was checked with AES, UPS and MIES.

The clusters were generated using a laser evaporation source; a detailed description can be found elsewhere^47^. In brief, the second harmonic of a Nd:YAG laser (InnoLas DPSS, 532 nm, 100 Hz) is focused onto a rotating platinum target (99.95% purity, Alfa-Aesar), with each pulse being thermalized and extracted into the vacuum with a delayed pulse of helium gas (6.0 purity, Westfalen). Electrostatic lenses guide the clusters to a bender where the positively charged species are guided into a quadrupole mass spectrometer (QMS; Extrel, 16,000 a.m.u.). The cluster beam is then size selected and a single cluster size is deposited onto the MgO(100)/Mo(100) substrate. Retarding field analyses ensure that the clusters have a kinetic energy of no more than 1 eV per atom for soft-landing conditions. The clusters are neutralized by electron tunnelling through the MgO film and the resulting current is recorded and integrated in order to calculate a cluster coverage. It is assumed that each cluster has unit charge. All TPR curves shown were performed on 9 × 10^12^ clusters. The majority of the pulsed valve experiments were also performed with this coverage and extrapolating data measured on lower coverages to 9 × 10^12^ did not produce any significant variations in the TOF.

### Cluster stability

Cluster stability at the relevant temperatures was ensured using a variety of techniques. For platinum clusters of various sizes, we have done repeated, consecutive, HD exchange or deuterium TPD experiments up to ∼320–350 K and observe no change in the desorption peak temperature or area. From this observation, it was concluded that the TPD results (Fig. 1 in the manuscript) indeed reflect true size effects. In addition, the pulse-to-pulse variations, seen in Supplementary Fig. 6B at 300 and 400 K for Pt_9_ and Pt_10_ (Supplementary Fig. 5), also reflect the stability of the clusters (against sintering, coalescence or fragmentation) under the reaction conditions of the isothermal (multi-cycle) experiments presented in this work. Any appreciable degree of sintering would be coupled with a very clear change in activity in both cases, which was not observed for the cluster sizes determined as being structure insensitive in a TPR run or at isothermal conditions.

Size-selected clusters have also been extracted from the experimental setup for stability studies on a silica support using nanoplasmonic sensing and transmission electron microscopy^53^. It was shown that after 21 h at 453 K, no sintering of size-selected samples (Pt_22_ and Pt_68_) is observed. In addition, Pt_68_ resists sintering at 533 K during the hydrogen oxidation reaction. STM results from size-selected Pd clusters show that on relatively strongly, supported binding boronitride film surfaces, annealing to 500 K for 5 min does not change the cluster size distribution (height histogram)^54^. The underlying principle of this stability is on the one hand the suppression of the Ostwald ripening through the relatively weak interaction of the transporting species (Pt-atoms) with the oxide support^54^ and the monodispersity of the cluster samples with the lack of the driving force for Ostwald ripening^53^. In fact, in these experiments, Ostwald ripening is only observed when reaching the Hüttig temperature of the cluster material, which for platinum is ∼650 K. The observed absence of Smoluchowski ripening can be understood when considering the relative large binding energies of the clusters with the support material. A reliable estimate of the energy that anchors the cluster to the underlying MgO(100) supporting surface is given by the calculated vertical BE (vBE), given as


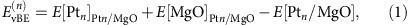


where *E*[Pt_*n*_/MgO] is the total energy of the structurally relaxed (energy optimized) ground state (lowest energy isomer) of the Pt_*n*_ cluster adsorbed on the MgO surface, and *E*[X] × _/MgO_ is the total energy of the isolated component (X=Pt_*n*_ or MgO) of the combined adsorption system, each calculated in the geometry that it assumes in the relaxed (optimized) configuration. The calculated values for *E*_vBE_^(*n*)^ for the three cluster sizes considered in this work, *n*=9, 10 and 13, are: *E*_vBE_^(*n*)^=6.96, 7.65 and 9.09 eV, respectively. These values reflect strong bonding of the clusters to the MgO(100) surface, inhibiting Smoluchowski ripening processes.

### TPR and H/D exchange experiments

The temperature programmed reaction (TPR) experiments were performed by dosing 0.4 H_2_ (5.0 purity, Air Liquide) molecules per MgO surface atom (1 cm^2^=2.25 × 10^15^ surface atoms) followed by 0.4 C_2_H_4_ (3.5 purity, Westfalen) ethylene molecules per MgO surface atom, using a calibrated molecular beam doser at a crystal temperature of 100 K. The crystal was then positioned ∼5 mm away from a skimmer leading to a differentially pumped QMS (Balzers QMA 430, Liechtenstein) chamber. A temperature ramp was then applied (Eurotherm 2408) and the ion signal of 30 *m*/*z* (ethane parent peak) was recorded.

**Figure 6:**
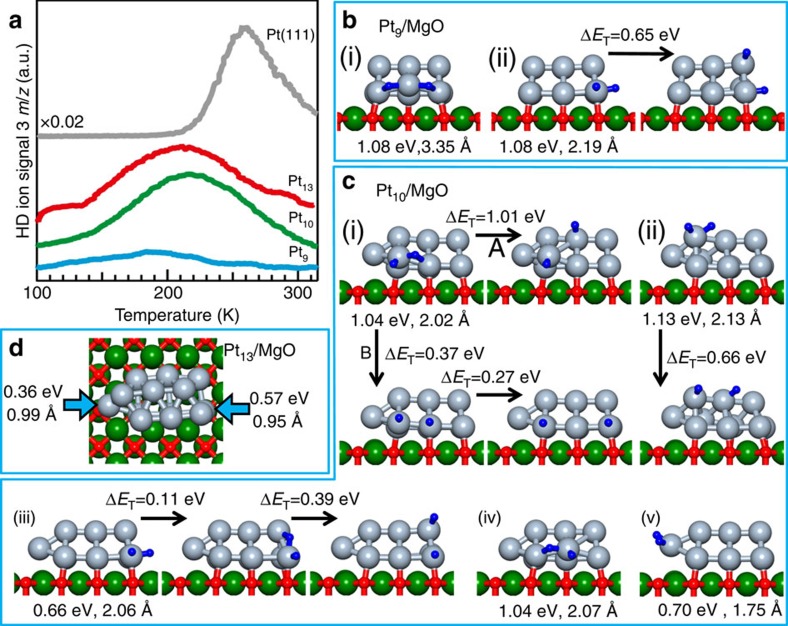
Hydrogen adsorption on supported platinum clusters. (**a**) TPR measurements of the H/D exchange on Pt_*n*_/MgO, *n*=9, 10, 13 and on Pt(111). (**b**,**c**) Calculated hydrogen dissociative adsorption at various sites, and diffusion on Pt_9_/MgO and Pt_10_/MgO, respectively. The pair of values under an adsorption configuration give the H_2_ adsorption energy and distance between the dissociated H atoms. Δ*E*_T_ denotes the activation barrier for diffusion between the corresponding H-atom configurations on the Pt cluster. (**d**) On Pt_13_/MgO, H_2_ dissociates at all sites except the ones marked by an arrow. Spheres colored green denote magnesium atoms, oxygen atoms are colored red, platinum atoms are coloured red, Pt atoms in grey, and hydrogen atoms are depicted by small blue spheres. The cluster configurations correspond to the lowest energy cluster-adsorption configuration on MgO(100).

Before executing the TPR measurements displayed in Fig. 1, we performed measurements and theoretical calculations pertaining to a key step of the HP ethylene hydrogenation mechanism, namely the dissociative adsorption of H_2_. To this end, we utilize isotopic H/D exchange (scrambling) TPR measurements. These HD-exchange experiments were performed in the same manner as described above for the other TPR measurements, except that a gas mixture of H_2_ and D_2_ (100.00%, Westfalen) was used where each partial pressure equaled 0.4 molecules per surface atom. A mass of 3 *m*/*z* was measured for these experiments. H/D exchange spectra have been recorded for a range of cluster sizes and the results for Pt_9_, Pt_10_ and Pt_13_ are displayed in Fig. 6a showing a limited activity for the Pt_9_ cluster compared with that of the larger clusters (peaking at *T*∼200 K), along with the measured record for the Pt(111) surface that shows onset of H/D exchange at a higher temperature and a peak centred at 260 K.

Hydrogen molecules are predicted from our DFT calculations to dissociatively adsorb on all Pt atoms of the bottom layer of Pt_9_ (Fig. 6b), but do not bind to top layer atoms of the cluster; the only way that an H atom may adsorb on the top layer is by diffusing (with an activation energy of 0.65 eV) from a bottom atom after dissociation, see Fig. 6b(ii) for details). The limited adsorption and dissociation of hydrogen on the Pt_9_ cluster correlate with the measured H/D exchange (Fig. 6a). On the other hand, dissociative H_2_ adsorption occurs readily at all sites of the bottom layer of the supported Pt_10_ and Pt_13_ clusters, with diffusion of the dissociated adsorbed hydrogen atoms between bottom layer sites entailing small barriers of the order of 0.1–0.3 eV, and interlayer diffusion requiring barriers that are as low as 0.40 eV. Unlike the case of the smallest cluster (Pt_9_), H_2_ dissociatively adsorbs on one of the second layer atoms of the Pt_10_ clusters (see Fig. 6c(ii), diffusing to a neighbouring upper layer atom with an activation energy of 0.66 eV), and the Pt_13_ cluster dissociatively adsorbs H_2_ at all sites (except one, see right arrow, Fig. 6d and Supplementary Fig. 1). The abundant sites for dissociative adsorption on both the bottom and top layers of the Pt_10_ and Pt_13_ clusters and the facile inter-site H-diffusion on these clusters correlate with the measured low-temperature H/D exchange signal (Fig. 6a).

### The pulsed molecular beam technique

The pulsed molecular beams technique used in our laboratory has been previously described^55^, and a brief overview will be given here. A piezo pulser, which allows for highly reproducible gas pulses, was filled with a 2-Torr background pressure of ethylene. From the time-resolved QMS response to a single ethylene pulse, it was determined that there are ∼10^14^ gas molecules per pulse giving a local pressure of ∼5 × 10^−7^ mbar. A deuterium background pressure of 2 × 10^−6^ mbar was established with a leak valve and the temperature of the crystal was increased to 300 K. The crystal was again placed ∼5 mm in front of the skimmer (same QMS as for the TPD experiments) and ethylene was pulsed onto the surface at a rate of 0.1 Hz. The ethane production was measured by monitoring the mass signal at 31 *m*/*z* with an oscilloscope (LeCroy Wave-runner 44Xi-A). Within the signal wave, we define a quasi-steady-state regime, where the ethane production maintains a constant value over the course of 80 ms. Approximately 20 pulses are then recorded and the quasi-steady-state signal is averaged and used to calculate the TOF. Supplementary Fig. 6A shows a representative average of pulses for Pt_11_. A region displaying steady-state ethane production (in grey) is then defined and the TOF calculated from this signal. Supplementary Fig. 6B shows the pulse to pulse signal for the same experiment displayed in Supplementary Fig. 6A. The calibration of the mass spectrometer was performed by determining the QMS signal of a saturated monolayer of CO on Pt(111) using the TPD technique and then determining the sensitivity factor between ethane and CO. It should be noted that although we measure ethane at *m*/*z*=31, the factor between the signal from equal amounts of pure ethane (*m*/*z*=30) and deuterated ethane, measured on the peak *m*/*z*=31, is roughly^56^ 2. As this error is systematic, no influence on the trend observed, or even the order of magnitude of the signal, occurs.

### Infrared reflection absorption spectroscopy

(Thermo Electron Corp. Nicolet FT-6700) was performed in single reflection mode with an external MCT-detector (Thermo Electron Corp., MCTA-TRS). At the experimental steps, a background spectrum was acquired by averaging 256 scans at a resolution of 4 cm^−1^ with the sample at ∼100 K. Ten Langmuir of CO were dosed and a sample spectra recorded with the same parameters as the background.

### First-principles computational methodology

To model the Pt_*n*_/MgO systems, with *n*=8, 9, 10, 13, we employed a four-layer MgO(100) slab with a calculational cell consisting of 7 × 6 unit cells; each layer consisted of 42 Mg and 42 oxygen atoms with the atoms in the bottom layer held stationary (at the experimental lattice constant of 4.21 Å) and the atoms in the other three layers allowed to relax to the optimal atomic arrangement. The experiments in this paper were performed for MgO films with thickness of ten layers or more. Under these conditions, there is no effect of the underlying Mo(100) substrate on the behaviour at the top of the film. Indeed, the effect of the Mo substrate has been predicted early on theoretically^57^ to be limited to MgO films with a thickness of four to five layers; see also predicted thickness-dependent reactivities, where the catalytic activity of deposited metal clusters was predicted to depend on the thickness of the underlying metal-oxide film adsorbed on a metal substrate^58^,^59^. This has been verified experimentally in later reactivity studies of gold nanoclusters supported on MgO films of variable thicknesses^20^ and most convincingly through work function measurements^60^. The above-mentioned prediction and experimental verifications justify the procedure used in our calculations, where no effect of the underlying Mo substrate on the charging behaviour at the MgO/Pt nanocluster interface is considered.

The optimal (minimum total energy) configurations were determined (through unconstrained relaxation of the atomic positions, using a conjugate gradient search) when the calculated energy converged within 0.001 eV. The bare clusters were positioned on the MgO(100) surface, and their configuration, together with the positions of the atoms of the underlying substrate, was optimized via unconstrained relaxations as described above. The calculational supercell, which included the MgO(100) slab and a 24-Å-thick vacuum region, was periodically replicated. Once the optimal cluster structures have been determined the binding (adsorption) energies and atomic positions of the reactants (that is, the individual H_2_ and C_2_H_4_ molecules, as well as multiply adsorbed, and co-adsorbed, systems) were determined through total energy minimization. This relaxation procedure allows for adsorbate/reactant-induced structural relaxations of the underlying metal cluster and metal-oxide (MgO(100) substrate; this form of adsorbate-induced cluster-catalyst relaxation (illustrated in Supplementary Fig. 16) is a manifestation of a more general class of cluster relaxation processes termed fluxionality^18^, which includes also dynamical reaction-induced cluster fluxionality.

In the first-principles calculations of the reaction profiles (pathways), a reaction coordinate was judiciously chosen; typically, the reaction coordinate consists of the distance between two atoms of the reactant molecules (for example, an adsorbed H atom of a dissociated hydrogen molecule and the C atom of a reacting ethylene molecule); the reaction coordinate can in general be a combination of geometrical parameters (interatomic distances and/or angles). For each value of the reaction coordinate, the total energy of the system, calculated with density functional theory (DFT) was optimized through unconstrained relaxation of all of the other degrees of freedom of the system (reactants, other adsorbents, Pt cluster atoms and MgO slab atoms atoms). The reaction profiles (reaction paths) were obtained via repeating such calculations for a sequence of values of the chosen reaction coordinate. These calculations yield results that are the same as, or very close to, those obtained by other methods, for example, the nudged elastic band and variants thereof; see the ref. 13.

It is pertinent to note here that the structural relaxation following reactant adsorption and subsequent to each reaction step (using a conjugate gradient method) allows for distortions of the combined system (underlying support surface, adsorbed cluster and reactants) and leads to certain adsorption and reaction—induced structural rearrangements; for example, in some cases it can bring about transformation of the cluster geometry to a different structural isomeric form, as in the aforementioned example of dynamical reaction-induced cluster fluxionality (Supplementary Fig. 16). Global modifications of the structural motif of the adsorbed cluster/adsorbate system involve prohibitive activation energy barriers and are unlikely to occur as these transformations would be kinetically non-viable, particularly for the adsorption and reaction conditions considered in our studies that involve relatively low temperatures, ranging from below room temperature up to 400 K. Consequently, for kinetic reasons, it appears that relaxations involving local minima of the free-energy landscape of the combined catalytic system (rather than the global ones) are more effective here in steering the reaction pathway and determining the catalysed reaction barriers.

All the aforementioned first-principles electronic structure calculations employed the DFT method as implemented in the VASP-DFT package, using a plane-wave basis with a kinetic energy cutoff of 400 eV, projector augmented wave (PAW) pseudopotentials^61^ and the Perdew, Burke & Ernzerhof (PBE) generalized gradient approximation for the exchange-correlation potential^62^,^63^,^64^,^65^,^66^. Γ- point sampling of the Brillouin zone was used.

## Additional information

**How to cite this article:** Crampton, A. S. *et al*. Structure sensitivity in the nonscalable regime explored via catalysed ethylene hydrogenation on supported platinum nanoclusters. *Nat. Commun.* 7:10389 doi: 10.1038/ncomms10389 (2016).

## Supplementary Material

Supplementary Information Supplementary Figures 1-36, Supplementary Note 1-2 and Supplementary References
