# Supplementary material for: Structure sensitivity in the nonscalable regime explored via catalysed ethylene hydrogenation on supported platinum nanoclusters
Source: Nat Commun. 2016 Jan 28;7:10389. doi: 10.1038/ncomms10389 (PMC4738346; doi:10.1038/ncomms10389)
Supplement: Supplementary Information — Supplementary Figures 1-36, Supplementary Note 1-2 and Supplementary References [file ncomms10389-s1.pdf]

Supplementary Figures

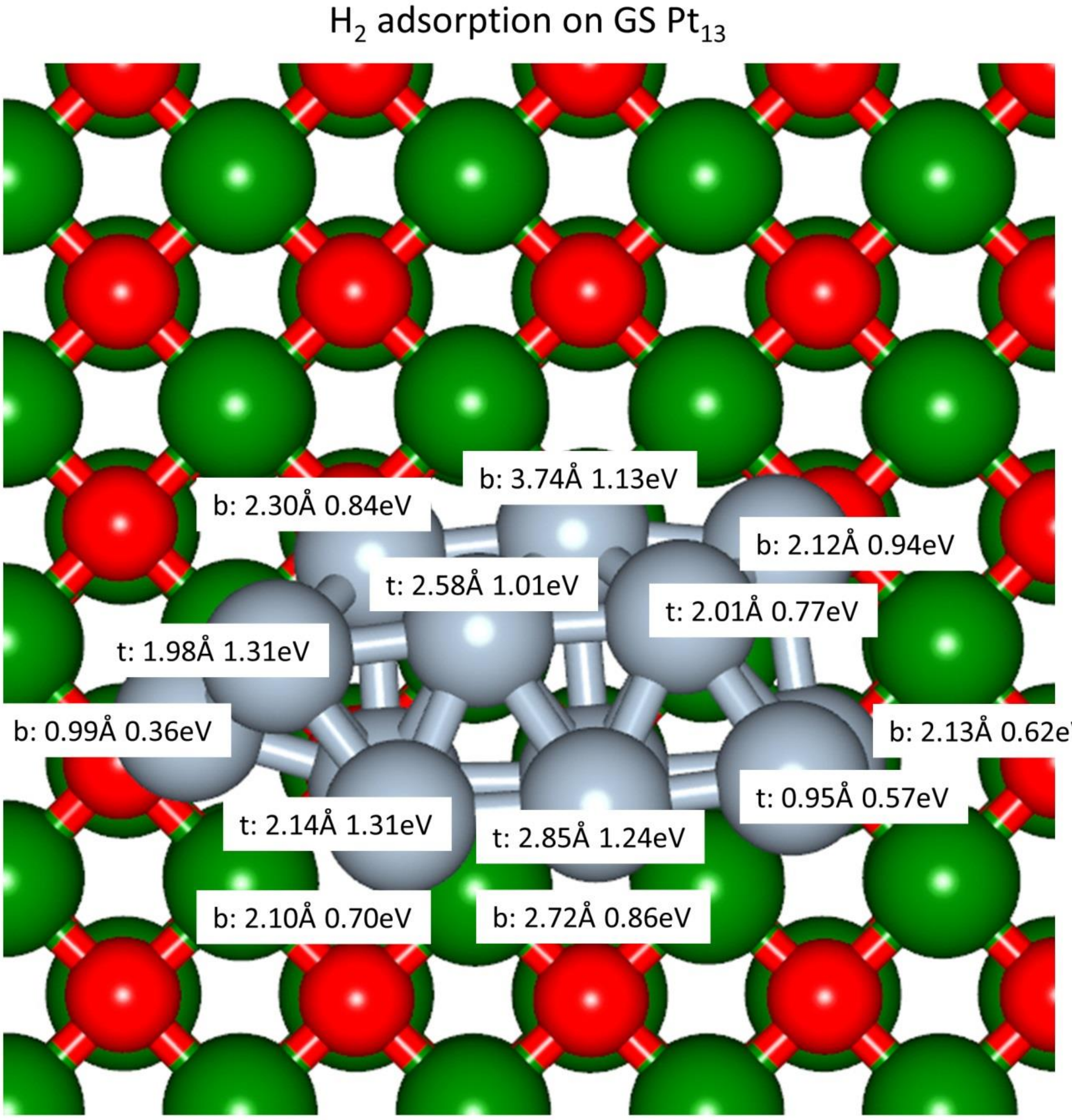

Supplementary Figure 1 | Hydrogen adsorption on a ground state supported Pt_{13} cluster. The platinuum atom are depicted by gray spheres adsorbed on MgO(100) (red speheres denote oxygen atoms, and green balls correspond to Mg atoms. The letters “b“ and “t“, for each of the cluster atoms designate “bottom“ and “top“ atoms of the cluster with respect to the cluster inteface with the MgO surface. Fot each of the sites we give the DFT-calculated distance between the two H atoms of the adsorbed H_2 molecules (with distances smaller than 1.0 Å corresponding to undissociated H_2 molecule, an dlarger values denoting a spontaneously dissociated molecule. The second value for each site gives the adsorption

energy of the molecule (in eV). It is evident that spontaneous dissociative adsorption occurs at all cluster sites except two – interestingly these two (H_2 molecular adsorption sites) are charcterized by a large excess electron density (see Figure. 2c of the main text and Supplementary figure 15).

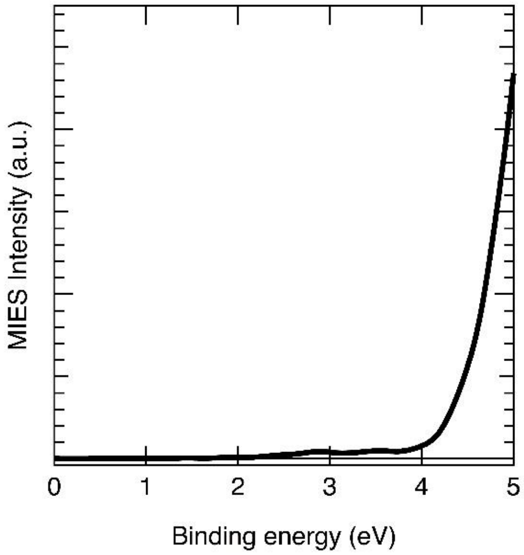

Supplementary Figure 2 | MIES spectrum of the MgO(100) thin film. The spectrum shows the valence band area of the MIES spectrum where no emission peak below 4 eV indicates the absence of F-center defects in the thin films.

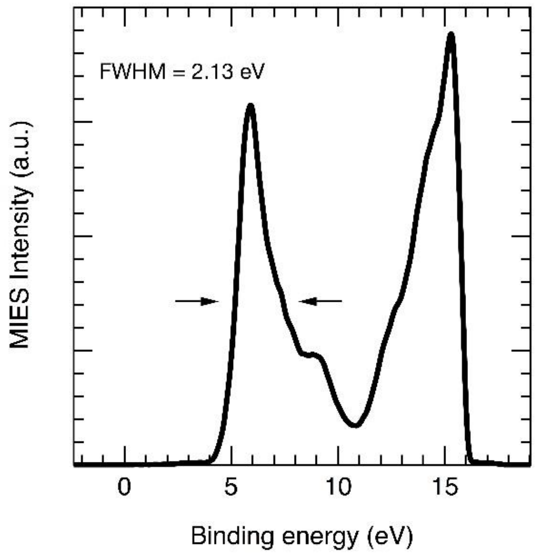

Supplementary Figure 3 | Complete MIES spectrum of MgO(100) thin film. The FWHM of the O2p peak is 2.13 eV, similar to that previously reported for a highly ordered film (see Ref. 51 of the main text).

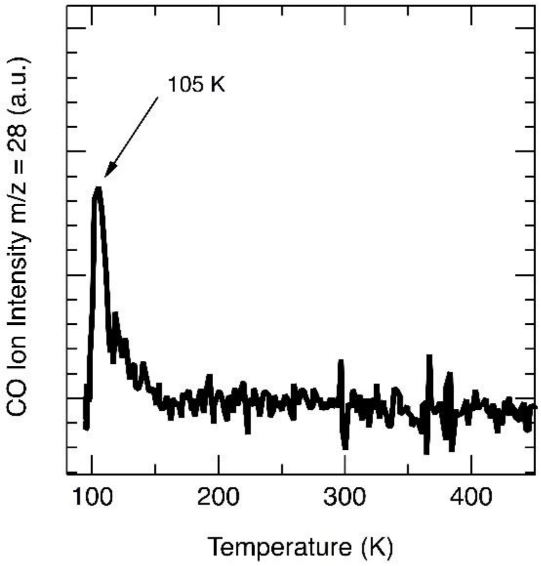

Supplementary Figure 4 | CO TPD from the MgO(100) thin film. One CO molecule per surface atom was dosed at 100 K and a temperature ramp of 2 K/s applied. The peak at 100 K is from a small amount of possible grain boundaries, corners and steps

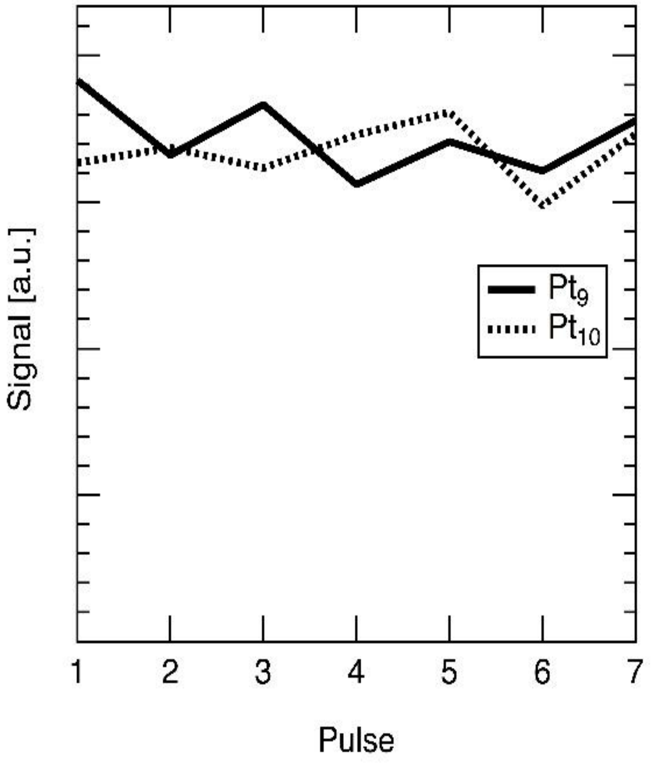

.

Supplementary Figure 5 | Pulse to pulse signal at 400 K from Pt_9 and Pt_{10}. The conditions were exactly the same as used for the catalytic experiments at 300 K (see Fig. 5a).

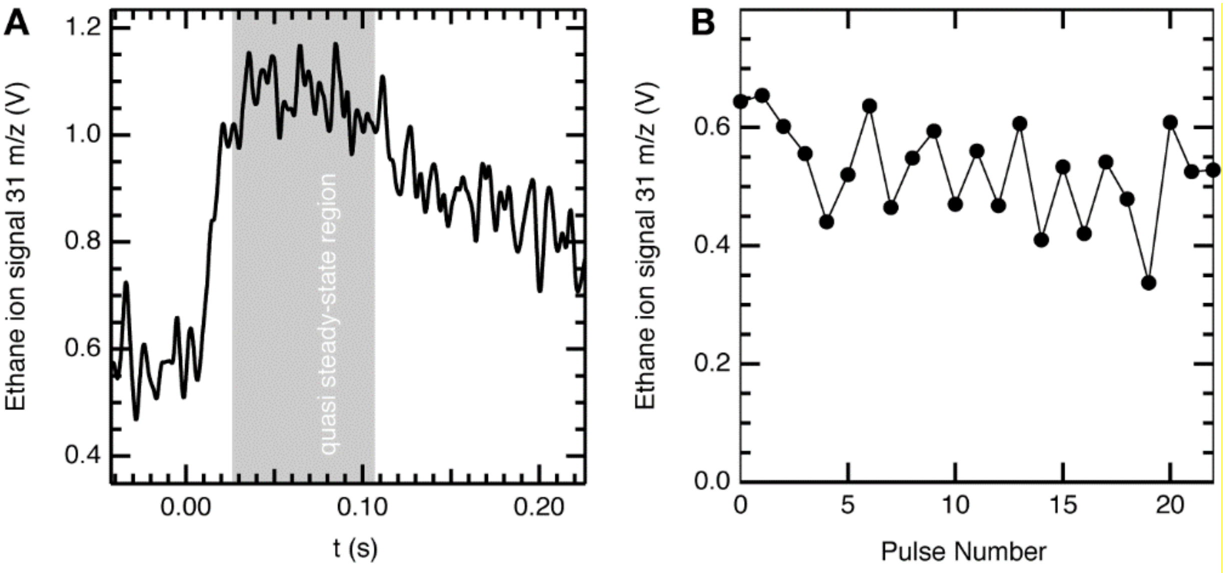

Supplementary Figure 6 | Representative pulsed molecular beam data for Pt_{11}. (A) Average ethane signal (31 m/z) of 20 pulses for Pt_{11} measured at 300 K. The gray, shaded area depicts the quasi steady state region (approx. 80 ms) where the turn over frequency was calculated. **(B)** Pulse to pulse ethane signal calculated for the same experiment as **A**.

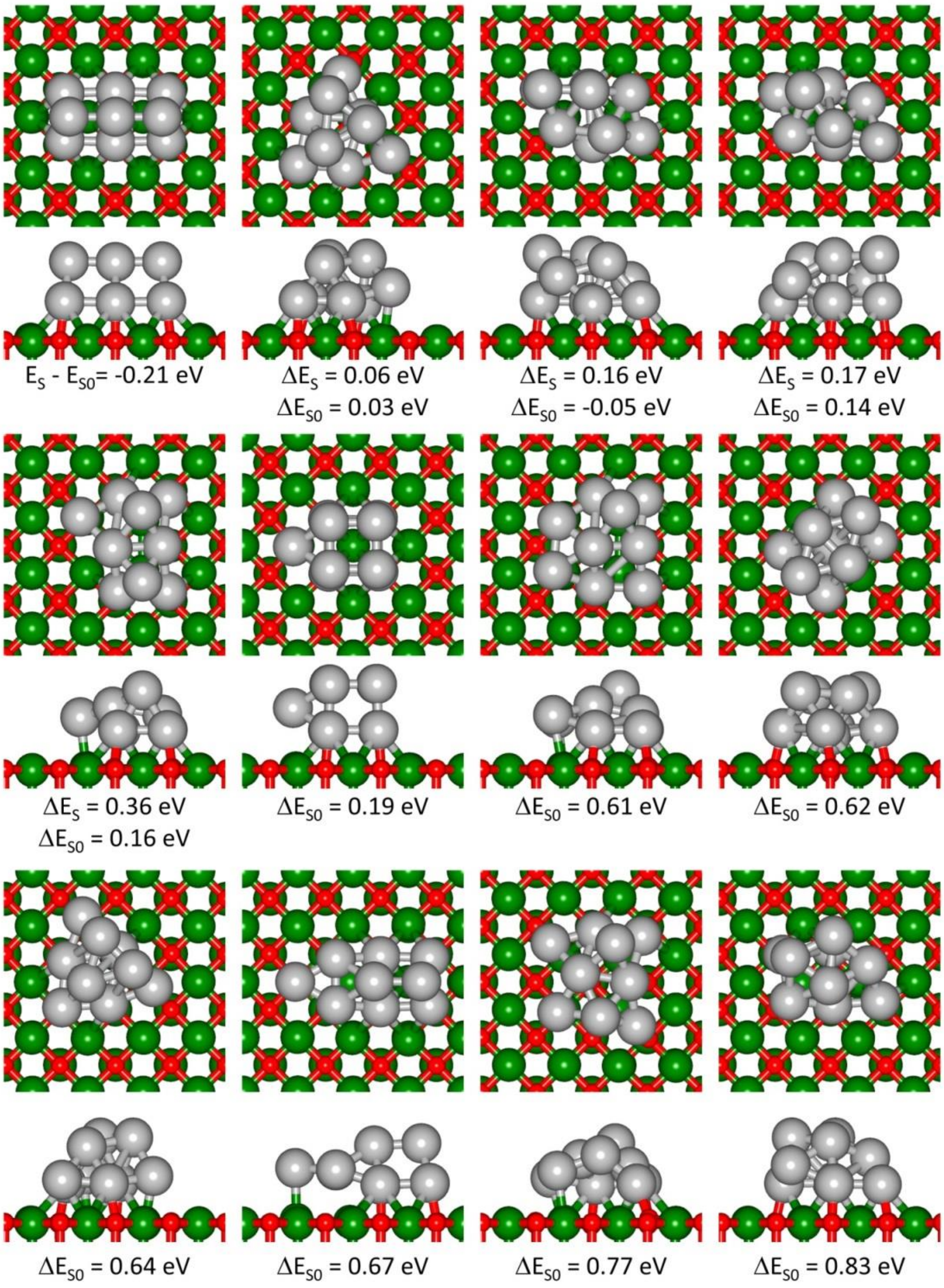

Supplementary Figure 7 | Ground state and higher –energy structural isomers for Pt_9/MgO(100). For the ground state (lowest energy) configuration (leftmost structure in the first row) E_s (E_{s0}) denote the energies calculated with (without, i.e. taking s=0)) spin optimization; in the calculations with spin optimization it has been found that the spin state of the bare adsorbed platinum clusters (Pt_n, n= 9,10, 13) is $N_\uparrow$-$N_\downarrow$=2 (where N_s is the number of electrons with spin s = ↑ or ↓). ΔE_s and ΔE_{s0} give the difference between the total energy of the isomer and the ground state, calclulated with (ΔE_s) and without (ΔE_{s0}) spin, repectively; ΔE_s is given for the first 5

isomers only. For each structure we give top (above) and side (below) views. Pt - gray spheres, Mg - green and O – red.

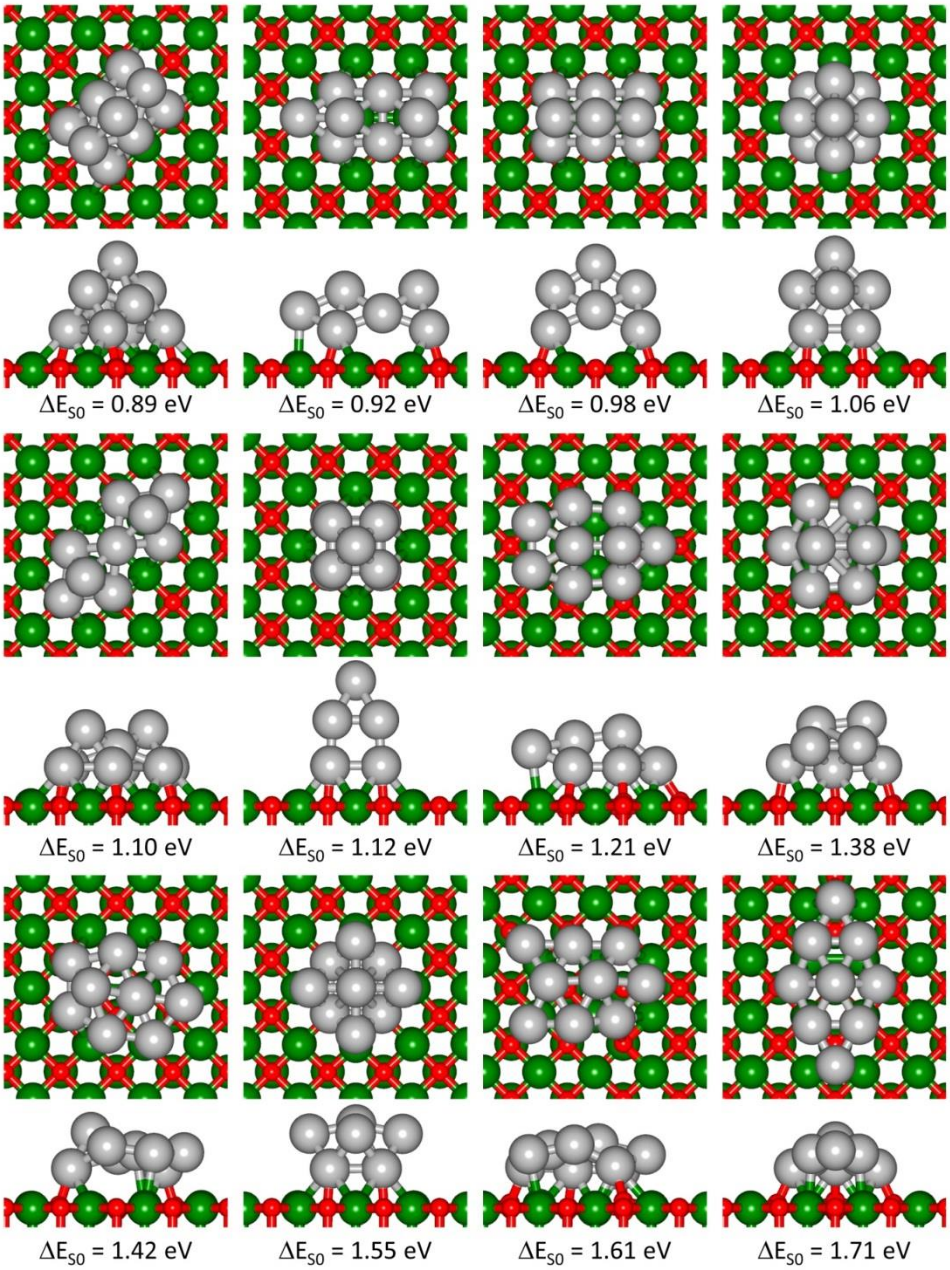

Supplementary Figure 8 | Ground state and higher –energy structural isomers for Pt_9/MgO(100). ΔE_{s0} gives the difference between the total energy of the isomer and the ground state (left-most structure in the first row of Supplementary Figure 7, calclulated without (ΔE_{s0}) spin, repectively. For each structure we give top (above) and side (below) views. Pt - gray spheres, Mg - green and O – red.

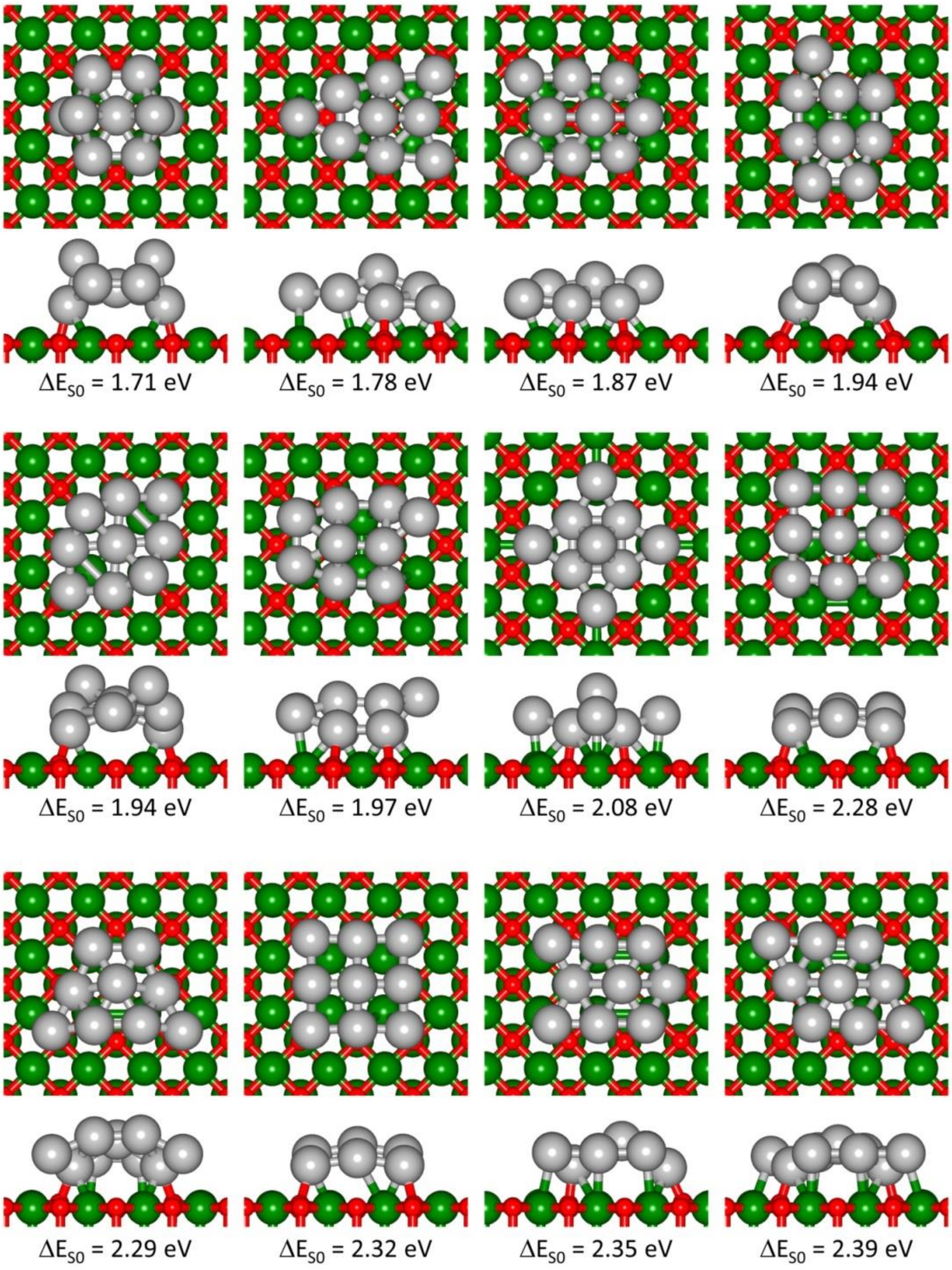

Supplementary Figure 9 | Ground state and higher –energy structural isomers for Pt_9/MgO(100). ΔE_{s0} gives the difference between the total energy of the isomer and the ground state (left-most structure in the first row of Supplementary Figure 7), calclulated without (ΔE_{s0}) spin, repectively, For each structure we give top (above) and side (below) views. Pt - gray spheres, Mg - green and O – red.

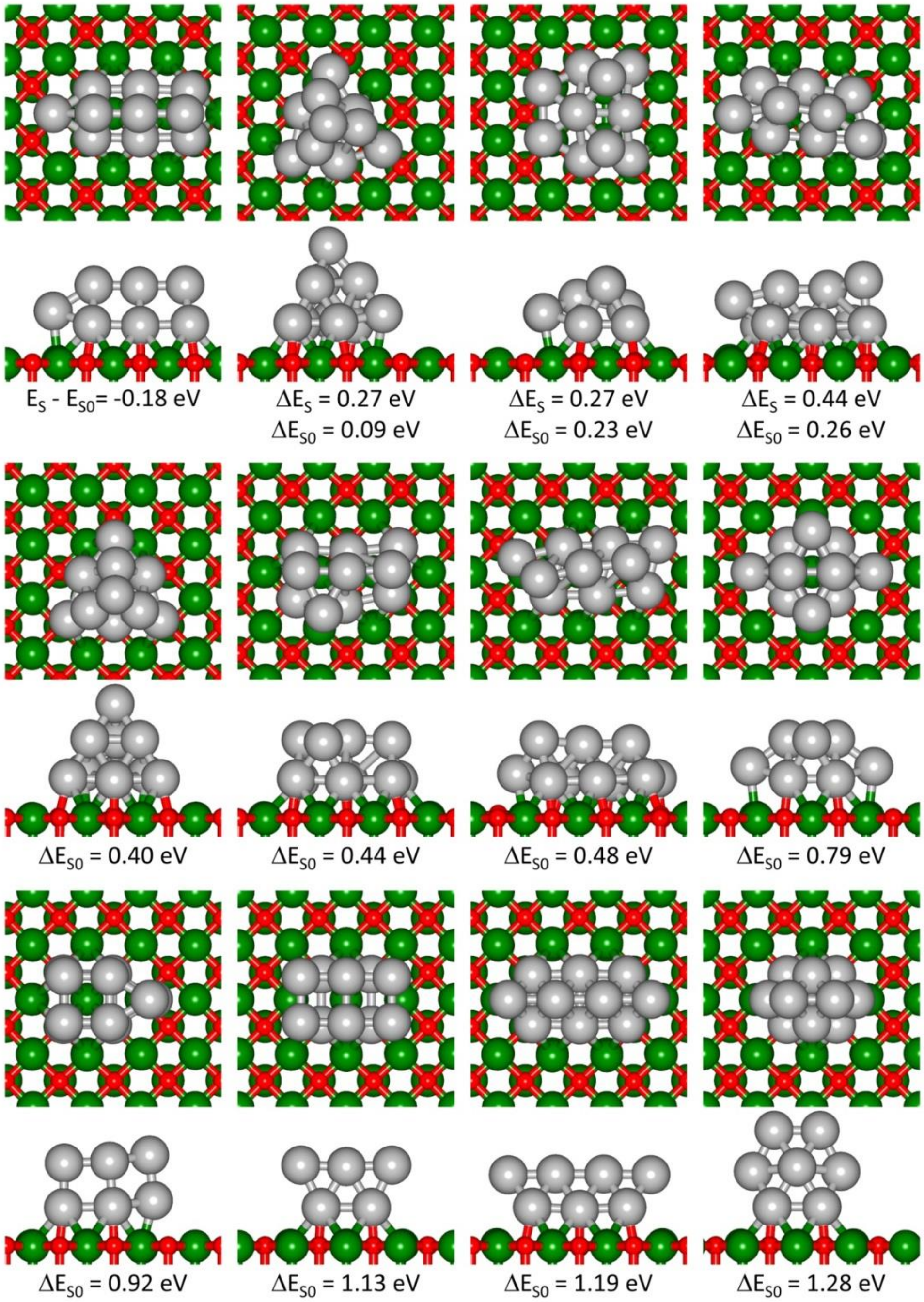

Supplementary Figure10 | Ground state and higher –energy structural isomers for Pt_{10}/MgO(100). For the ground state (lowest energy) configuration (leftmost structure in the first row) E_s (E_{s0}) denote the energies calculated with (without, i.e. taking s=0)) spin optimization. ΔE_s and ΔE_{s0} give the difference between the total energy of the isomer and the ground state, calclulated with (ΔE_s) and without (ΔE_{s0}) spin, repectively; ΔE_s is given for the first 4 isomers only. For each structure we give top (above) and side (below) views. Pt - gray spheres, Mg - green and O – red.

S

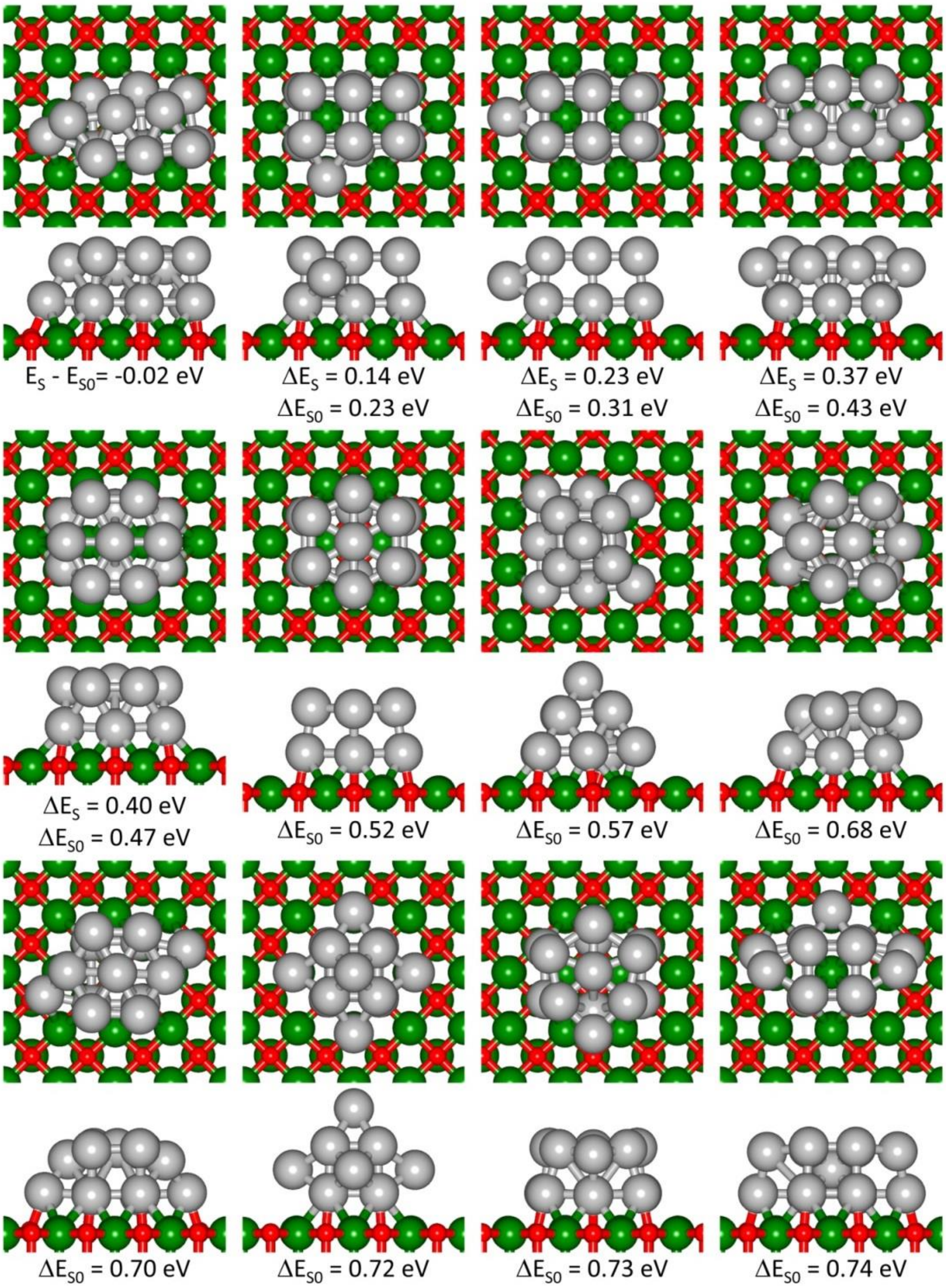

Supplementary Figure 11 | Ground state and higher –energy structural isomers for
Pt_{13}/MgO(100). For the ground state (lowest energy) configuration (leftmost structure in the first row) E_s (E_{s0}) denote the energies calculated with (without, i.e. taking s=0)) spin optimization. ΔE_s and ΔE_{s0} give the difference between the total energy of the isomer and the ground state, calclulated with (ΔE_s) and without (ΔE_{s0}) spin, repectively; ΔE_s is given for the first 4 isomers only. For each structure we give top (above) and side (below) views. Pt - Gray spheres, Mg - green and O – red.

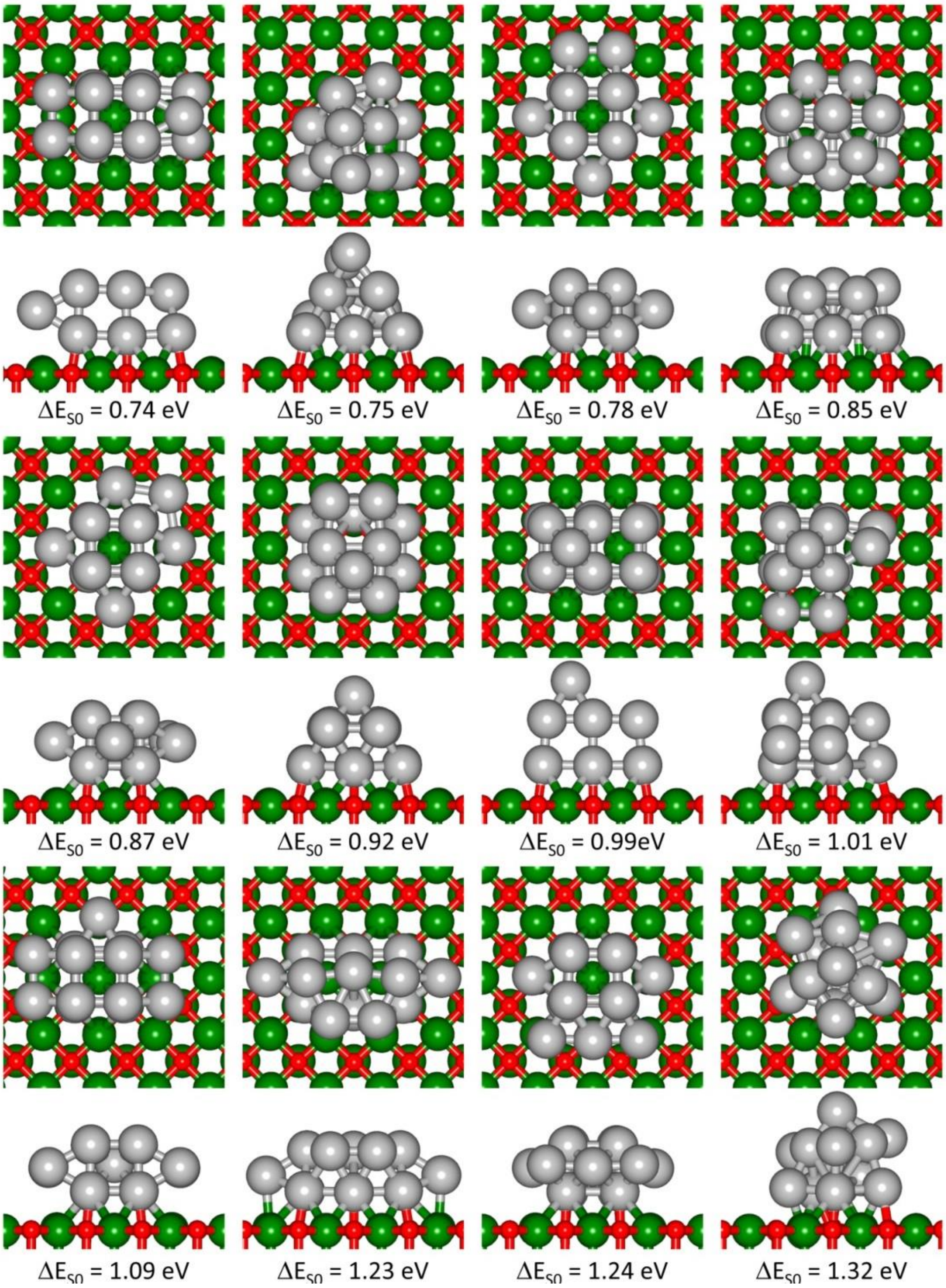

Supplementary Figure 12 | Ground state and higher –energy structural isomers for Pt_{13}/MgO(100). ΔE_{s0} gives the difference between the total energy of the isomer and the ground state (left-most structure in the first row of Figure S11, calclulated without (ΔE_{s0}) spin, repectively, For each structure we give top (above) and side (below) views. Pt - gray spheres, Mg - green and O – red.

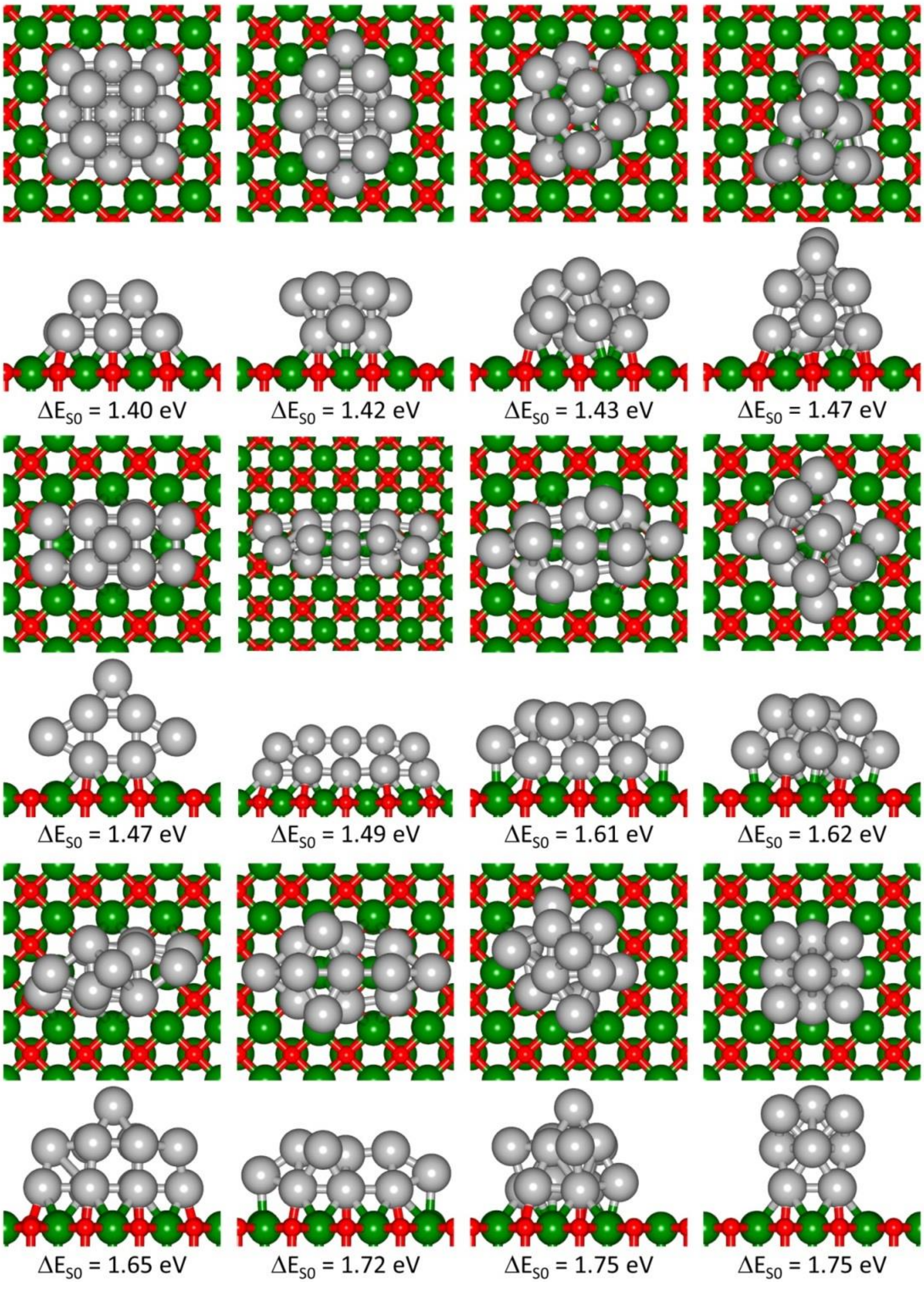

Supplementary Figure 13 | Ground state and higher –energy structural isomers for Pt_{13}/MgO(100). ΔE_{s0} gives the difference between the total energy of the isomer and the ground state (left-most structure in the first row of Supplementary Figure 11, calclulated without (ΔE_{s0}) spin, repectively, For each structure we give top (above) and side (below) views. Pt - gray spheres, Mg - green and O – red.

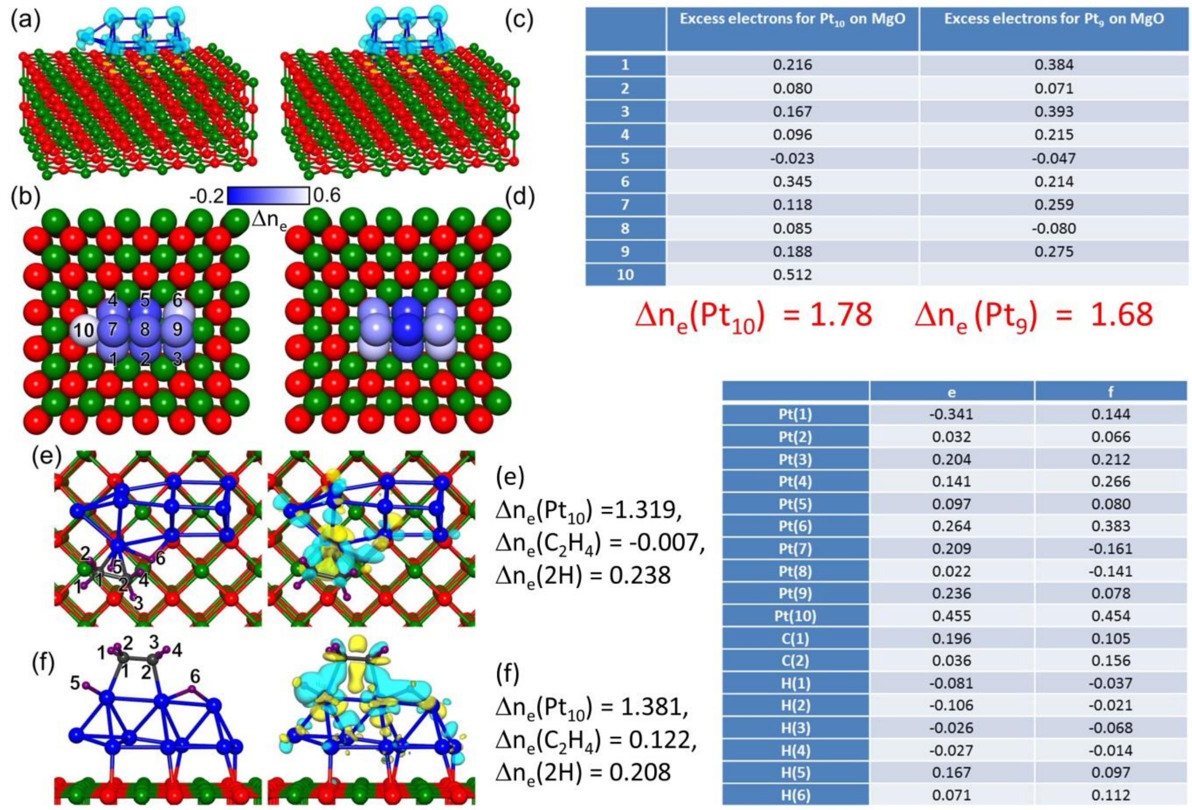

	Excess electrons for Pt_{10} on MgO	Excess electrons for Pt_9 on MgO
1	0.216	0.384
2	0.080	0.071
3	0.167	0.393
4	0.096	0.215
5	-0.023	-0.047
6	0.345	0.214
7	0.118	0.259
8	0.085	-0.080
9	0.188	0.275
10	0.512	

$\Delta n_e(Pt_{10}) = 1.78$ $\Delta n_e(Pt_9) = 1.68$

	e	f
Pt(1)	-0.341	0.144
Pt(2)	0.032	0.066
Pt(3)	0.204	0.212
Pt(4)	0.141	0.266
Pt(5)	0.097	0.080
Pt(6)	0.264	0.383
Pt(7)	0.209	-0.161
Pt(8)	0.022	-0.141
Pt(9)	0.236	0.078
Pt(10)	0.455	0.454
C(1)	0.196	0.105
C(2)	0.036	0.156
H(1)	-0.081	-0.037
H(2)	-0.106	-0.021
H(3)	-0.026	-0.068
H(4)	-0.027	-0.014
H(5)	0.167	0.097
H(6)	0.071	0.112

Supplementary Figure 14 | Optimal adsorption configurations and charge distribution on bare Pt_n / MgO (n= 9, 10) and co-adsorbed C_2H_4 +H_2 on Pt_{10}/MgO. In **a** and **c**, the blue and yellow contour hyper-surfaces correspond to excess (light blue) and deficient (yellow) charge distributions obtained as the difference between the total charges before and after adsorption of the clusters; these hypersurfaces are drawn such that the excess electronic (negative) charge inside the light blue hypersurface is 30% of the total electronic charge and the same for the positive charge inside the yellow hypersurfaces (for hypersurfaces corresponding to 50% of the excess negative (and positive) excess charges, see Fig. 2a and 2c, respectively, of the main manuscript text. Bader charge analysis is given in **b** and **d**, with lighter color corresponding to excess number of electrons (that is excess negative charge on the corresponding atom). For the values of the Bader charges are given in the table on the right. **(e,f)** Coadsorption of C_2H_4 and H_2 on Pt_{10}/MgO in the π (e) and di-σ (f) bonding modes. The adsorption geometries are shown on the left, and on the right we depict the bonding frontier orbitals of the adsorption system **(**the light blue and yellow denote different signs of the wave function); the σ-type Pt-C bonds are clearly seen in **f** (note the directed wave function contours on the right). In both **e** and **f**, atoms 5 and 6 are the proximal

dissociated coadsorbed H atoms. For the values of the Bader charges are given in the table on the right.

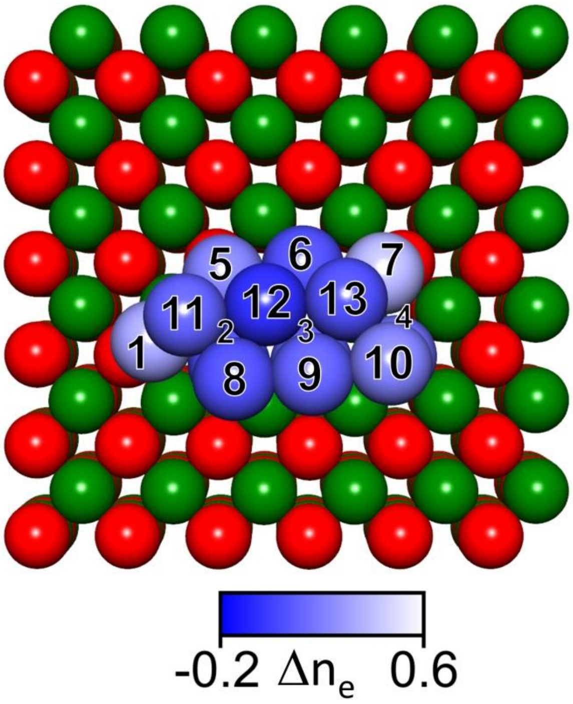

	Excess electrons for Pt_{13} on MgO
1	0.341
2	0.052
3	0.130
4	0.171
5	0.212
6	0.099
7	0.363
8	0.066
9	0.101
10	0.222
11	0.145
12	-0.038
13	0.058

$\Delta n_e\ (Pt_{13})_{Bader} = 1.922$

Supplementary Figure 15 | Optimal adsorption geometry of Pt_{13}/MgO and the Bader charge analysis. Lighter color corresponding to excess number of electrons (that is excess negative charge on the corresponding atom). For the values of the Bader charges are given in the table on the right. The excess electronic (negative) and positive charge hypersurfaces for the cluster are given in Fig. 2e of the main manuscript.

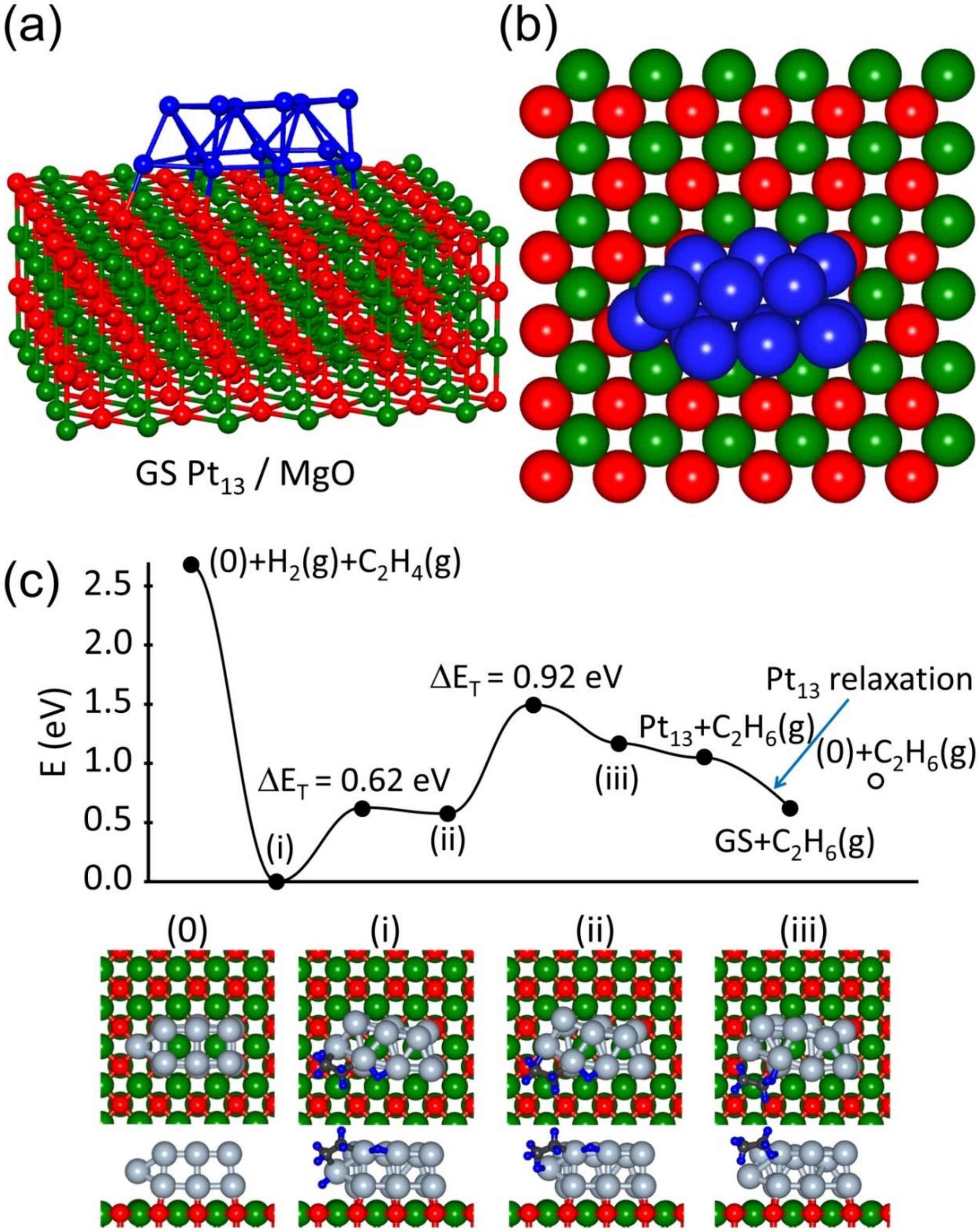

Supplementary Figure 16 | Adsorption and reaction induced structural fluxionality (**a,b**) side (**a**) and top (**b**) views of the ground state structure of Pt_{13}/MgO(100). (c) Fluxional transformation from an initial configuration (0) having a higher-in-energy isomer structure – see 2nd isomer in the top row of Supplementary Figure 11, with total energy (calculated without spin optimization) ΔE_{s0} = 0.31eV above the energy of the ground state, GS, isomer displayed in a&b of the current figure. This transformation is induced by the coadsorption of C_2H_4 and dissociated H_2, see configuration (i). Overcoming the two activation barriers

(0.66eV and 0.92 eV), the product at the end of the ethane (C_2H_6(g))-producing hydrogenation reaction contains a magnesia–supported Pt_{13} cluster with the GS structure (see a&b above) with a total energy lowering of the fully relaxed Pt_{13}/MgO system energy of 0.31 eV (compare the energy of the states GS + C_2H_6(g) denoted by the rightmost filled dot in the energy pathway in (**c**) with that of the state marked by the rightmost open dot, corresponding to (0) + C_2H_6(g), where (0) is the bare initial higher-in=energy isomer shown on the left of the bottom row of (**c**).

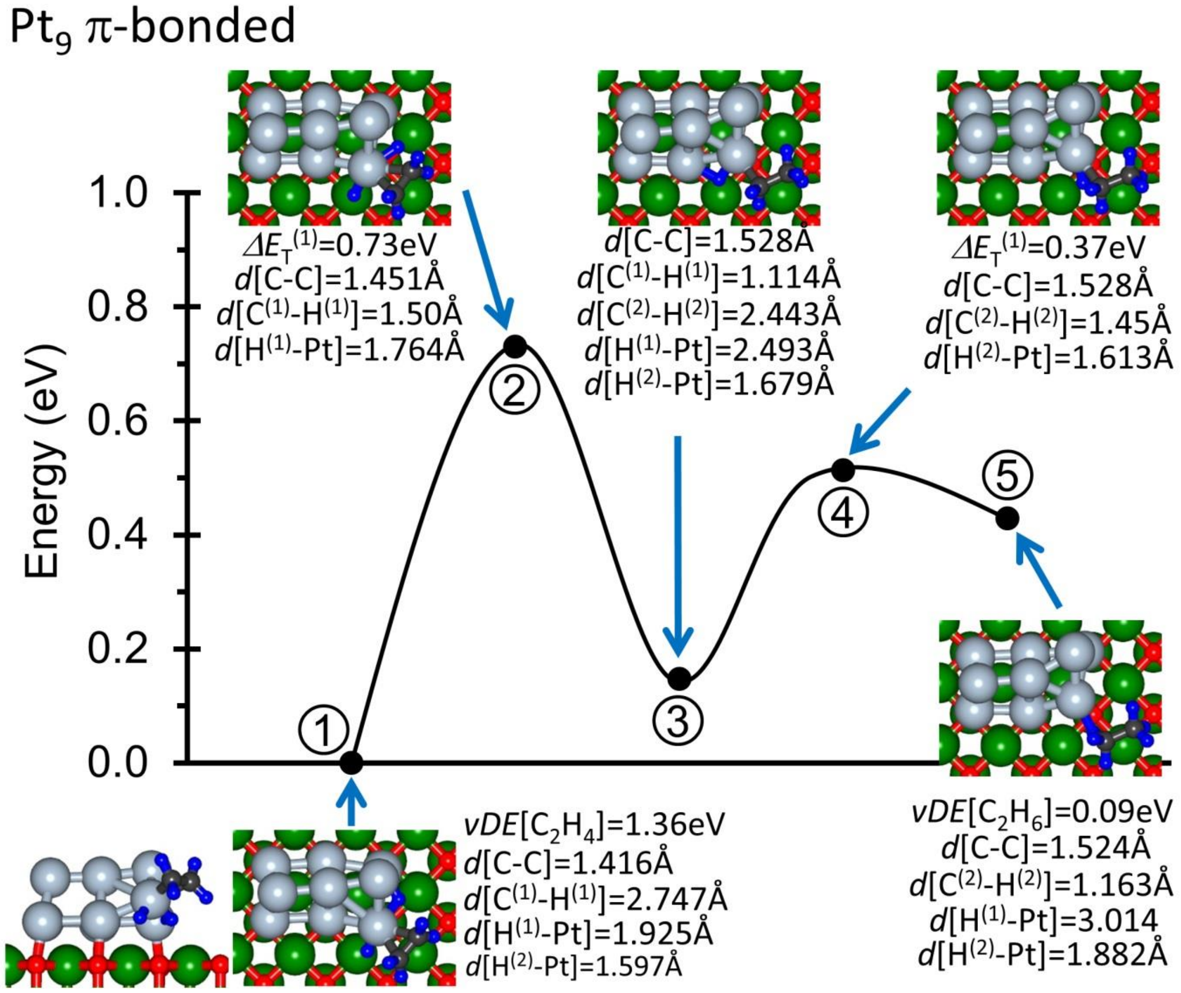

	d[C(1)-C(2)] (Å)	d[C(1)-H(1)] (Å)	d[C(1)-Pt] (Å)	d[H(1)-Pt] (Å)	d[C(2)-H(2)] (Å)	d[C(2)-Pt] (Å)	d[H(2)-Pt] (Å)
①	1.416	2.747	2.184	1.925	2.438	2.128	1.597
②	1.451	1.50	2.238	1.764	2.512	2.097	1.611
③	1.528	1.114	2.768	2.493	2.443	2.055	1.679
④	1.530	1.099	2.948	2.810	1.45	2.180	1.613
⑤	1.524	1.100	3.260	3.014	1.163	2.575	1.882

Supplementary Figure 17 | The lowest activation energy pathway for ethylene hydrogenation reaction on Pt_9/MgO. The reaction starts from a π-bonded C_2H_4 molecule coadsorbed with a dissociated H_2 molecule. The reaction follows the HP mechanism, with a first barrier of 0.73 eV. The geometric parameters along the reaction path are shown.

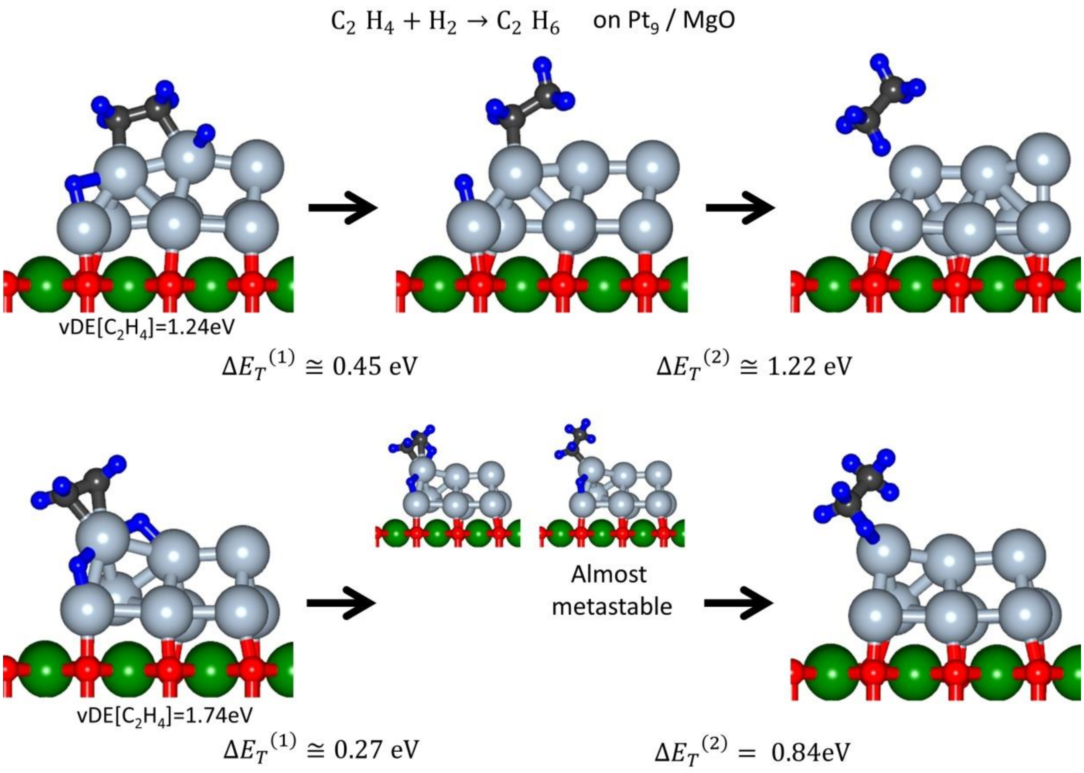

Supplementary Figure 18 | Higher-activation-barrier reactions on Pt_9/MgO(100). Upper row di-σ bonded, and bottom row π-bonded ethylene; H_2 does not spontaneously dissociate on top (second layer) of the adsorbed Pt_9 cluster, and H-diffusion from the interfacial layer entails an activation barrier.

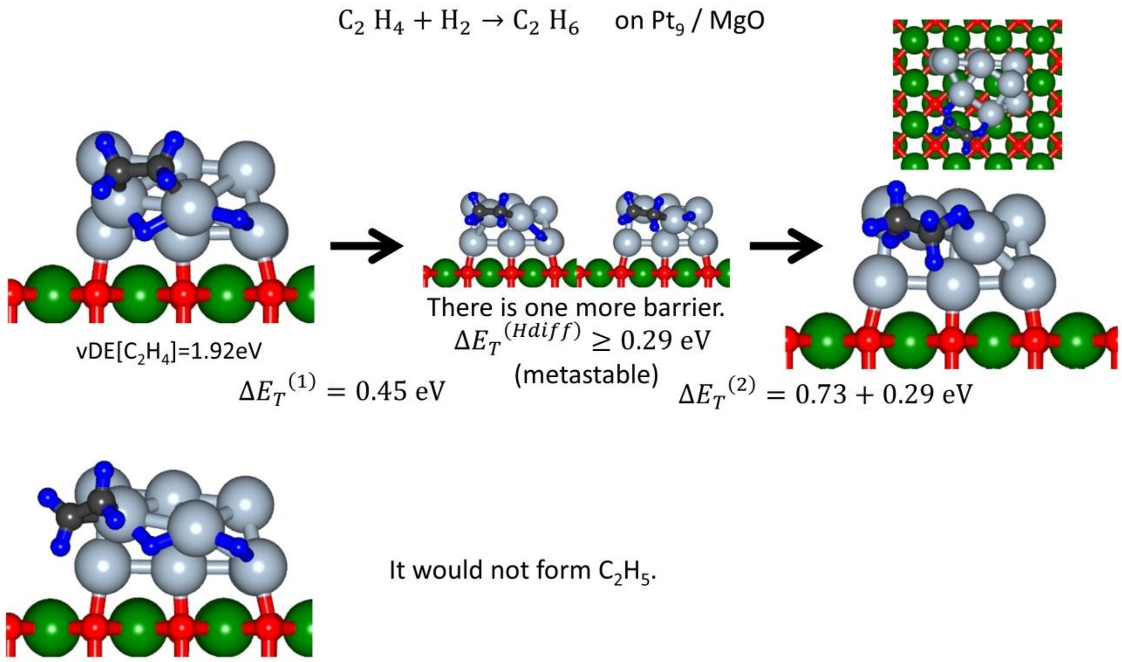

Supplementary Figure 19 | Higher-barrier ethylene hydrogenation reactions on Pt₉/MgO(100). **I**n the bottom row the approach of the H atom to the adsorbed ethylene is accompanied by a repulsive interaction**.**

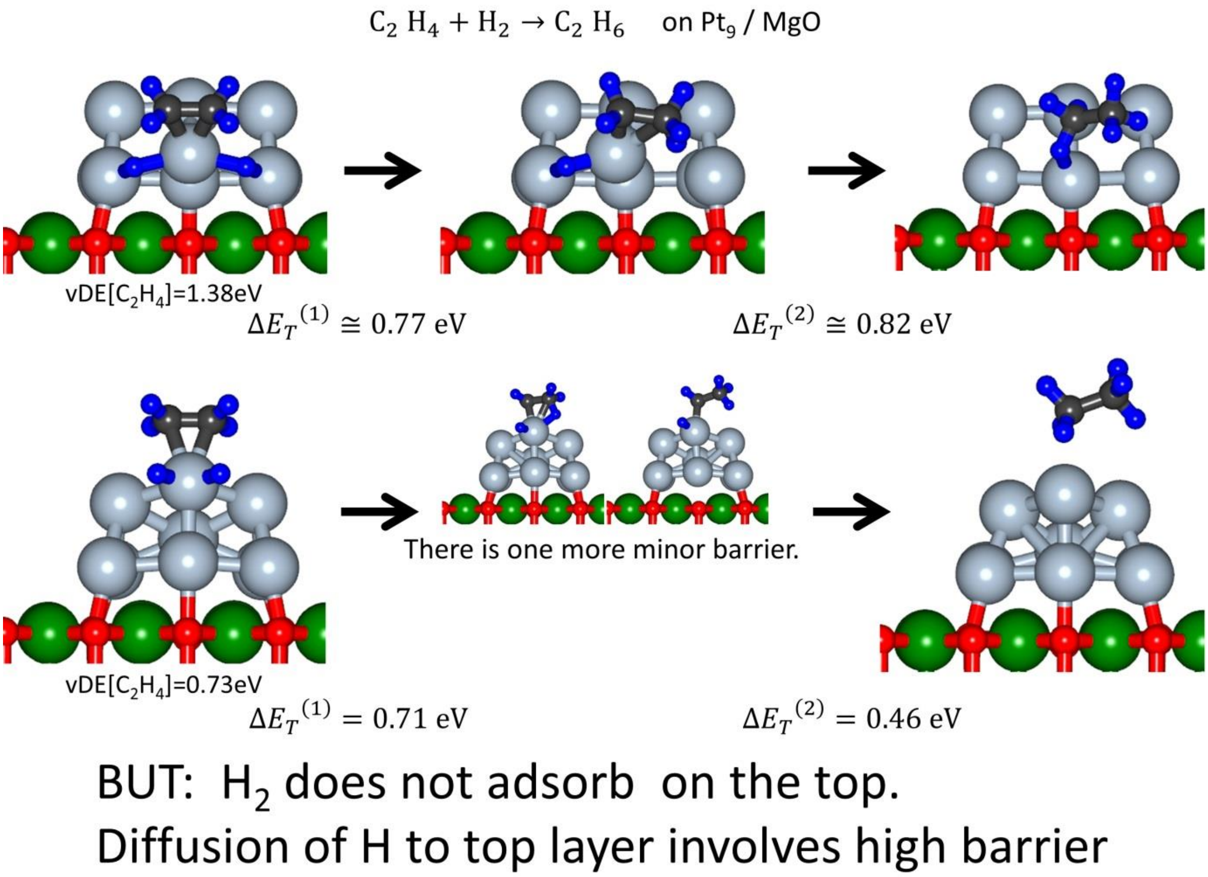

Supplementary Figure 20 | Higher-barrier ethylene hydrogenation reactions on Pt_9/MgO(100). Reactions starting from π –bonded ethylene to the central atom of the second-layer ridge.

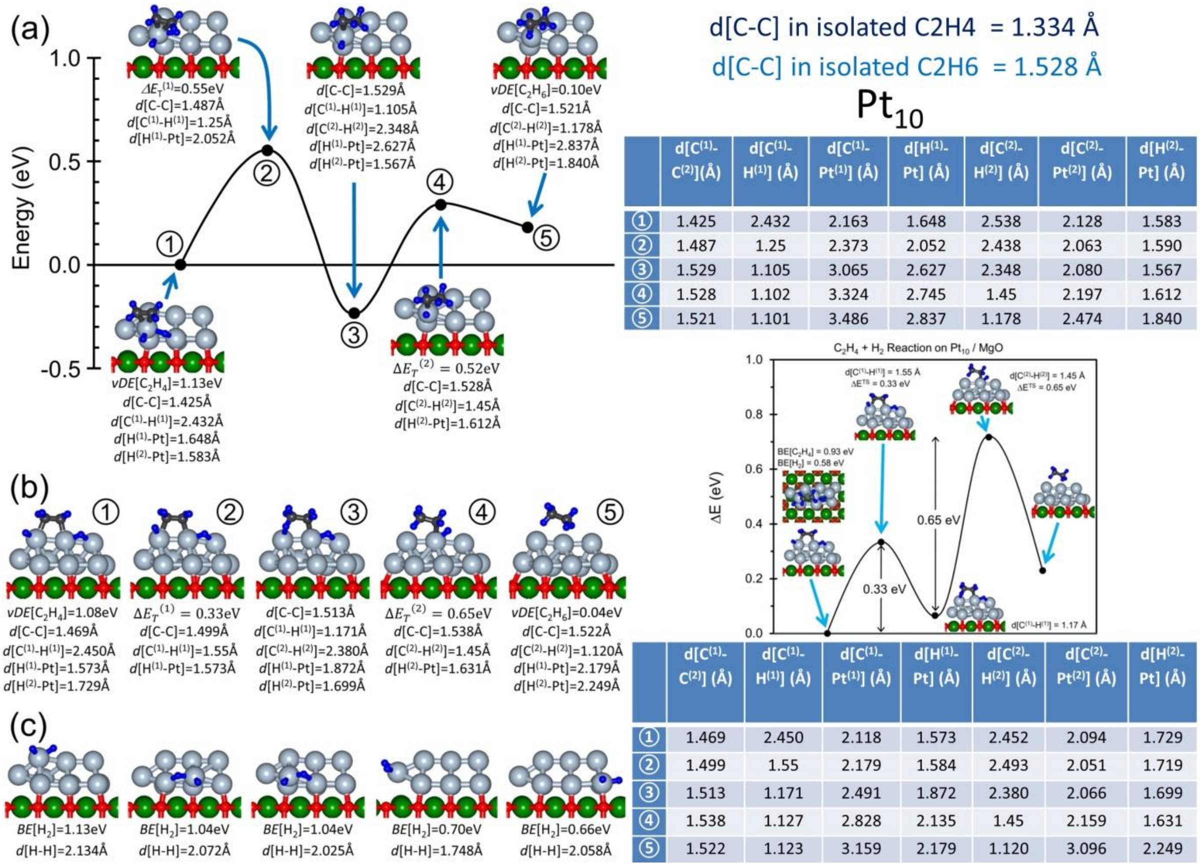

	d[$C^{(1)}$-$C^{(2)}$] (Å)	d[$C^{(1)}$-$H^{(1)}$] (Å)	d[$C^{(1)}$-$Pt^{(1)}$] (Å)	d[$H^{(1)}$-Pt] (Å)	d[$C^{(2)}$-$H^{(2)}$] (Å)	d[$C^{(2)}$-$Pt^{(2)}$] (Å)	d[$H^{(2)}$-Pt] (Å)
①	1.425	2.432	2.163	1.648	2.538	2.128	1.583
②	1.487	1.25	2.373	2.052	2.438	2.063	1.590
③	1.529	1.105	3.065	2.627	2.348	2.080	1.567
④	1.528	1.102	3.324	2.745	1.45	2.197	1.612
⑤	1.521	1.101	3.486	2.837	1.178	2.474	1.840

	d[$C^{(1)}$-$C^{(2)}$] (Å)	d[$C^{(1)}$-$H^{(1)}$] (Å)	d[$C^{(1)}$-$Pt^{(1)}$] (Å)	d[$H^{(1)}$-Pt] (Å)	d[$C^{(2)}$-$H^{(2)}$] (Å)	d[$C^{(2)}$-$Pt^{(2)}$] (Å)	d[$H^{(2)}$-Pt] (Å)
①	1.469	2.450	2.118	1.573	2.452	2.094	1.729
②	1.499	1.55	2.179	1.584	2.493	2.051	1.719
③	1.513	1.171	2.491	1.872	2.380	2.066	1.699
④	1.538	1.127	2.828	2.135	1.45	2.159	1.631
⑤	1.522	1.123	3.159	2.179	1.120	3.096	2.249

Supplementary Figure 21 | Low-barrier Ethylene hydrogenation pathways on Pt_{10}/MgO. Geometric details of the reaction pathways of (a) π-bonded ethylene and (b) di-σ bonded ethylene, as well as dissociative adsorption of H_2 (in (c)).

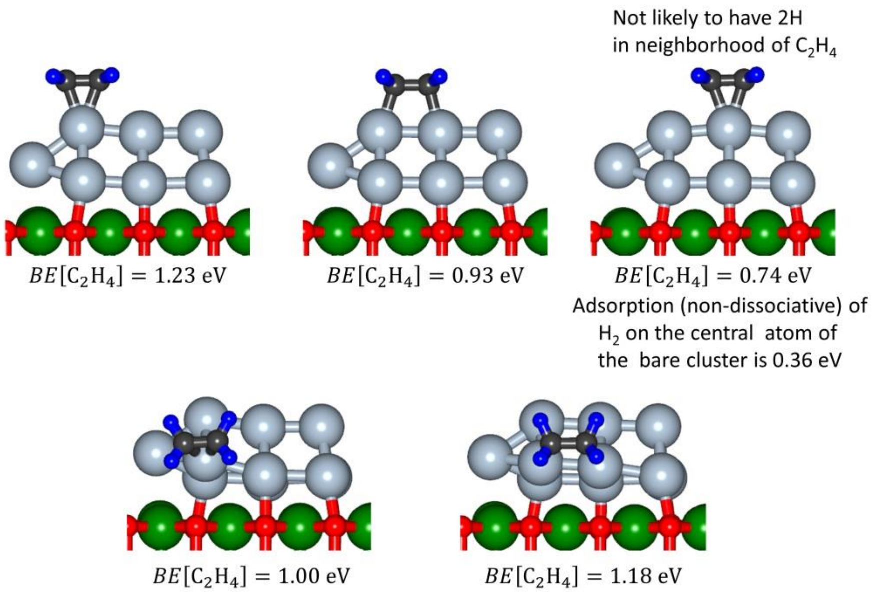

Supplementary Figure 22 | Selected Ethylene adsorption sites on Pt_{10}/MgO.

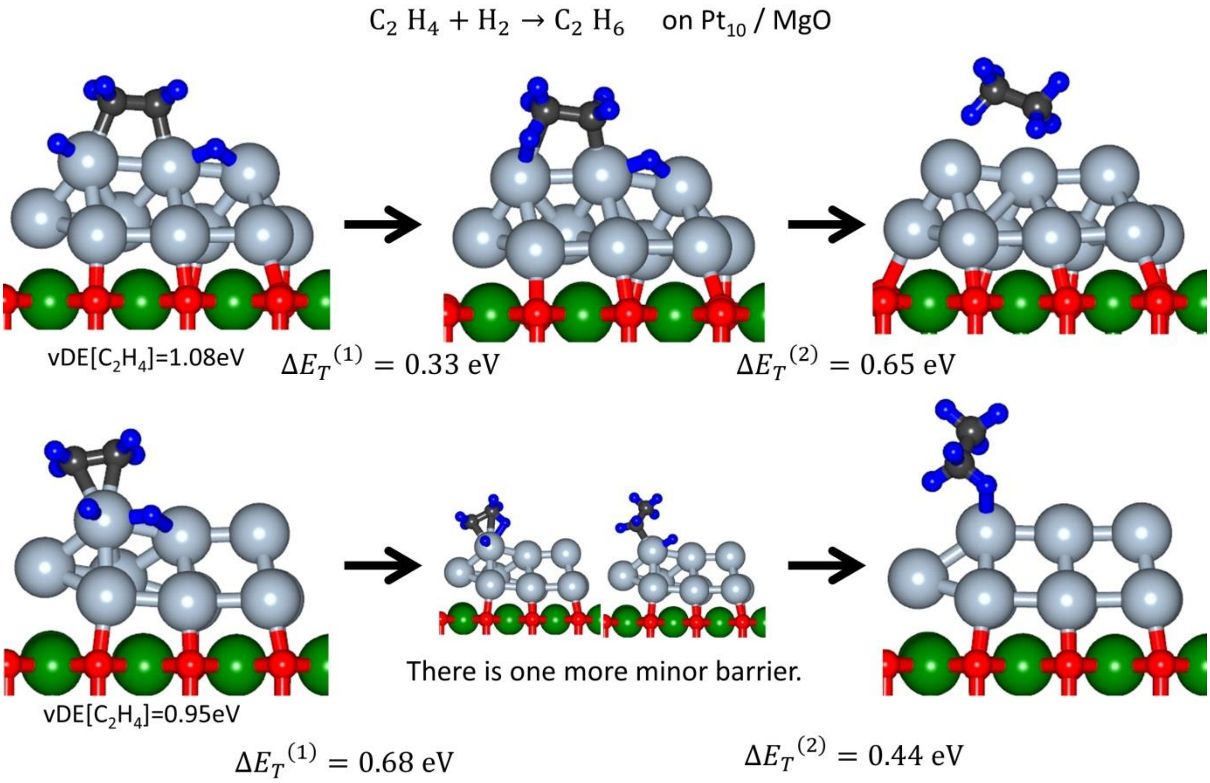

Supplementary Figure 23 | Relatively low-barrier ethylene hydrogenation pathways on Pt_{10}/MgO.

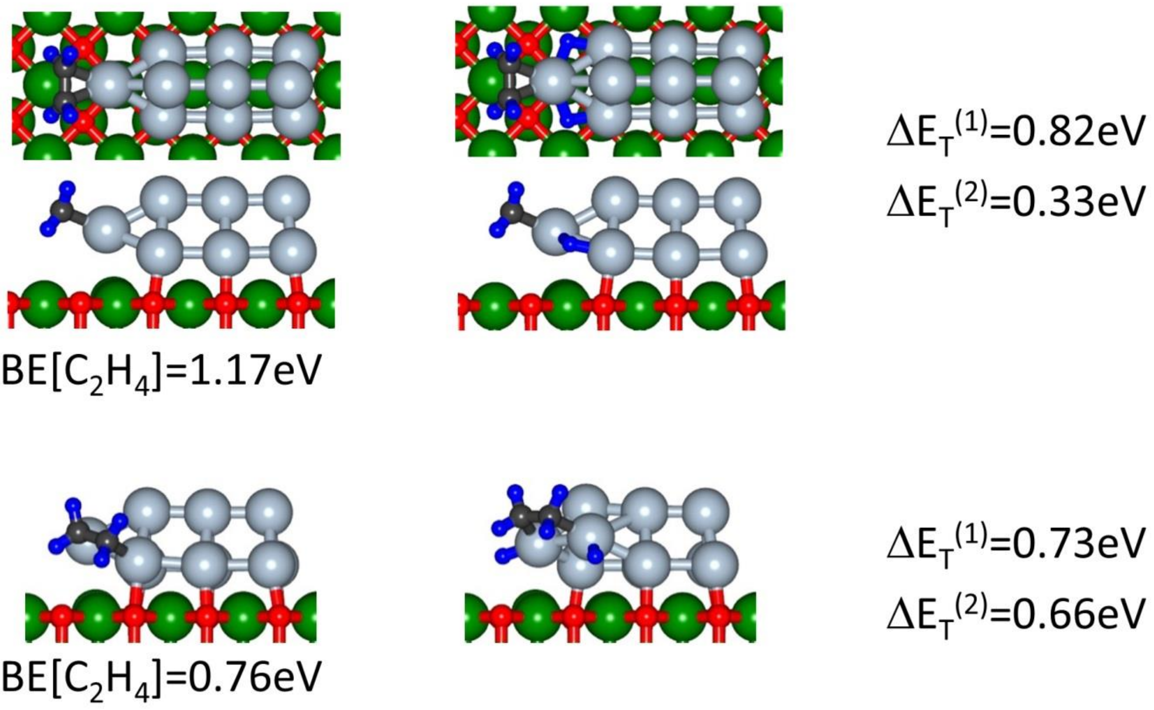

Supplementary Figure 24 | Higher energy ethylene hydrogenation sites.

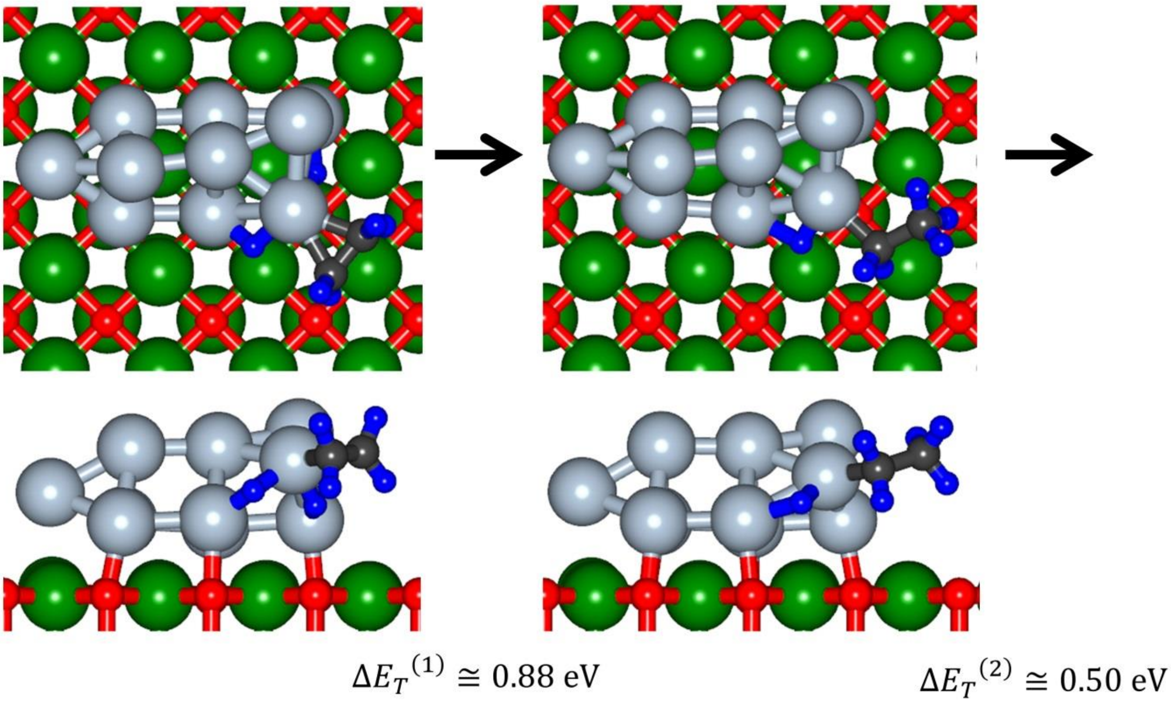

FIGURE S25: A higher energy ethylene hydrogenation site.

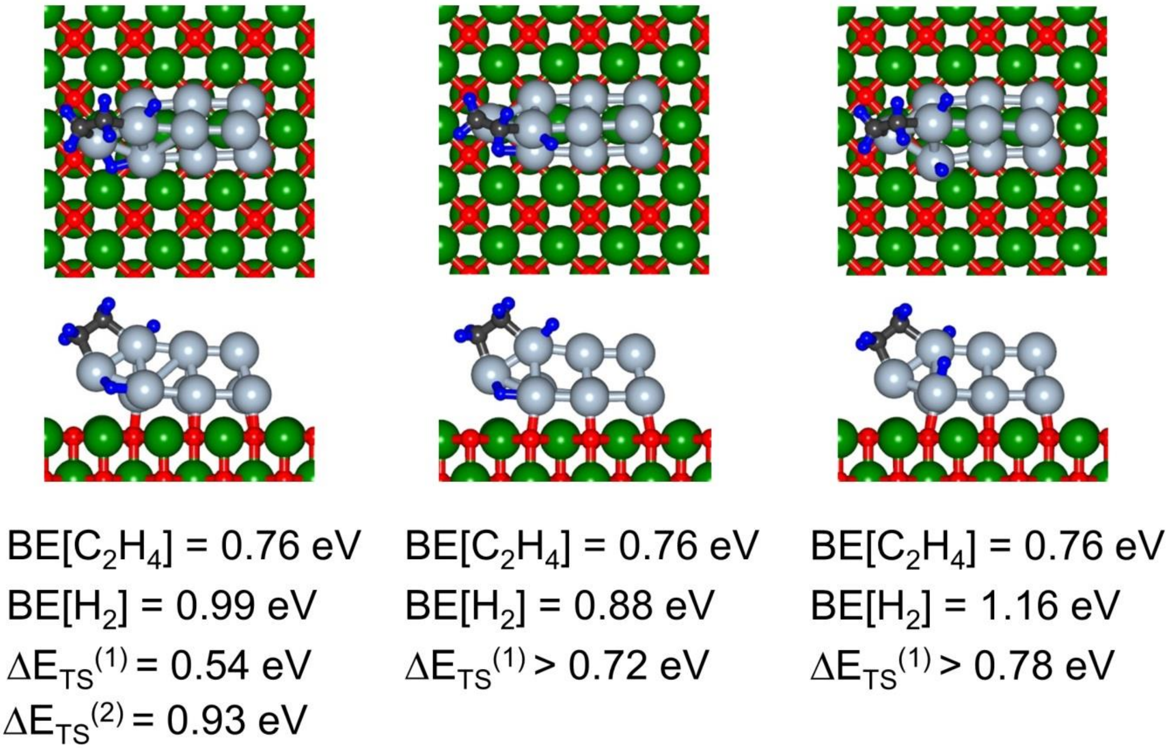

Supplementary Figure 26 | Additional higher energy ethylene hydrogenation sites.

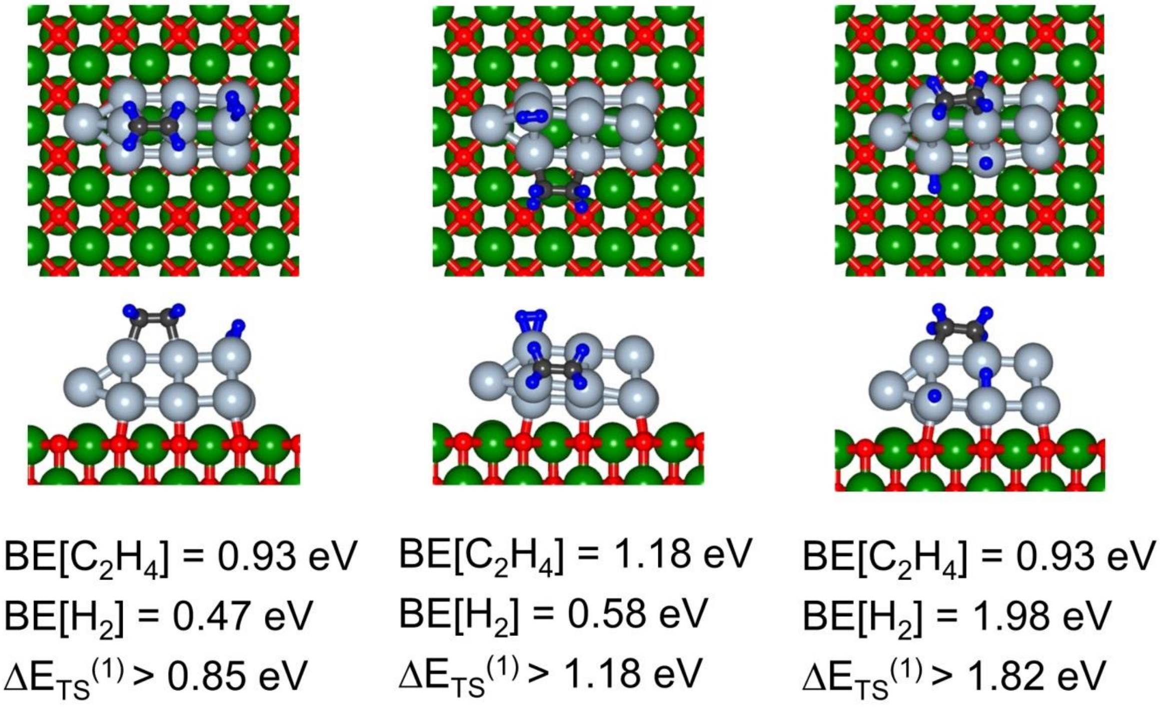

Supplementary Figure 27 | Even higher-barrier ethylene hydrogenation sites.

Supplementary Note 9: Ethylene Hydrogenation on Pt_{13}/MgO

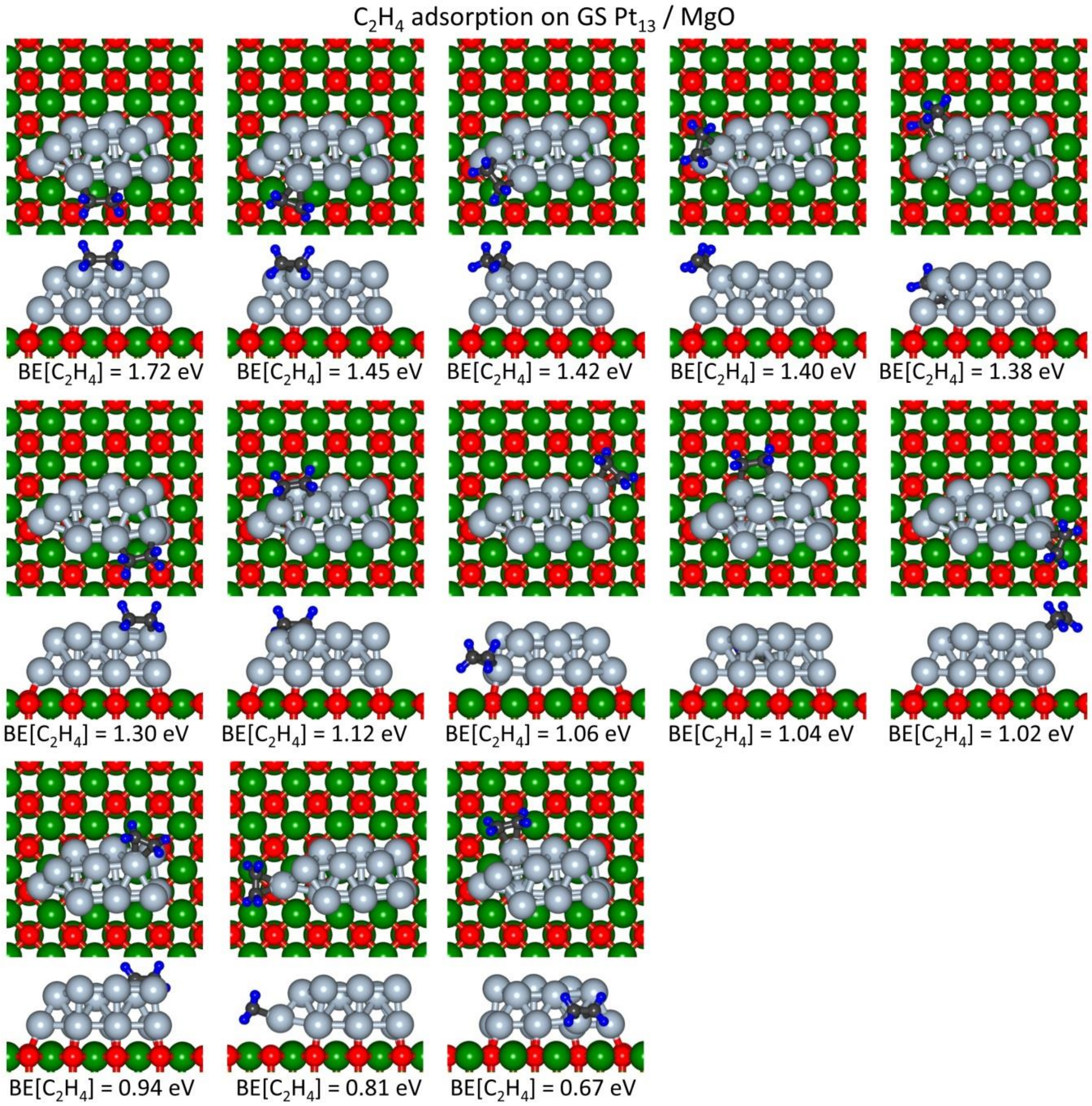

Supplementary Figure 28 | Binding sites of ethylene on the ground-state Pt_{13}/MgO system. The binding energies (BE) were obtained with the use of DFT calculations.

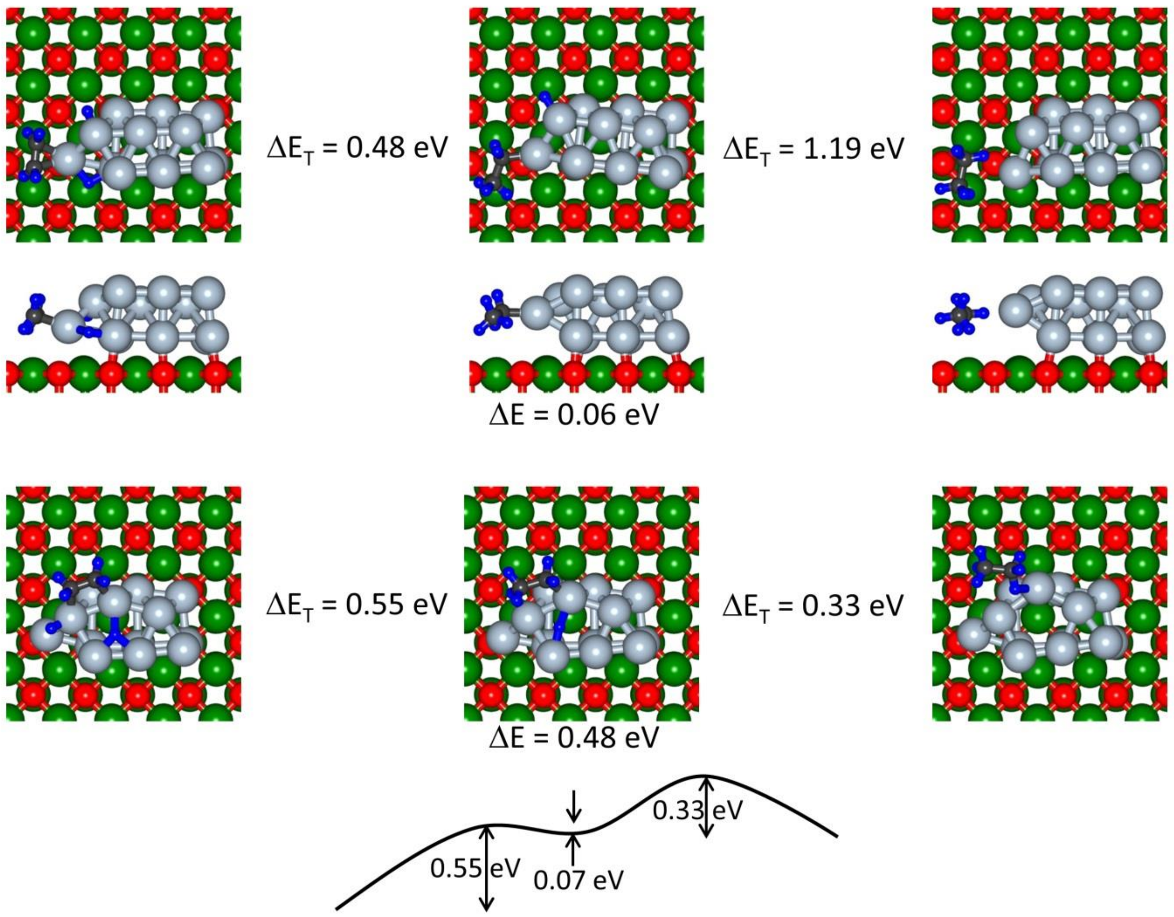

Supplementary Figure 29| Low and high barrier pathways of ethylene hydrogenation on Pt_{13}/MgO. Low-barrier (bottom row, starting from di-σ bonded C_2H_4 coadsorbed with dissociated H_2) and high-barrier (top-row, starting from π-bonded C_2H_4 coadsorbed with dissociated H_2) pathways of ethylene hydrogenation on Pt_{13}/MgO. ΔE_T = denotes the activation barrier, and ΔE gives the energy of the local minimum after the first activation barrier relative to the starting (initial) configuration (on the left).

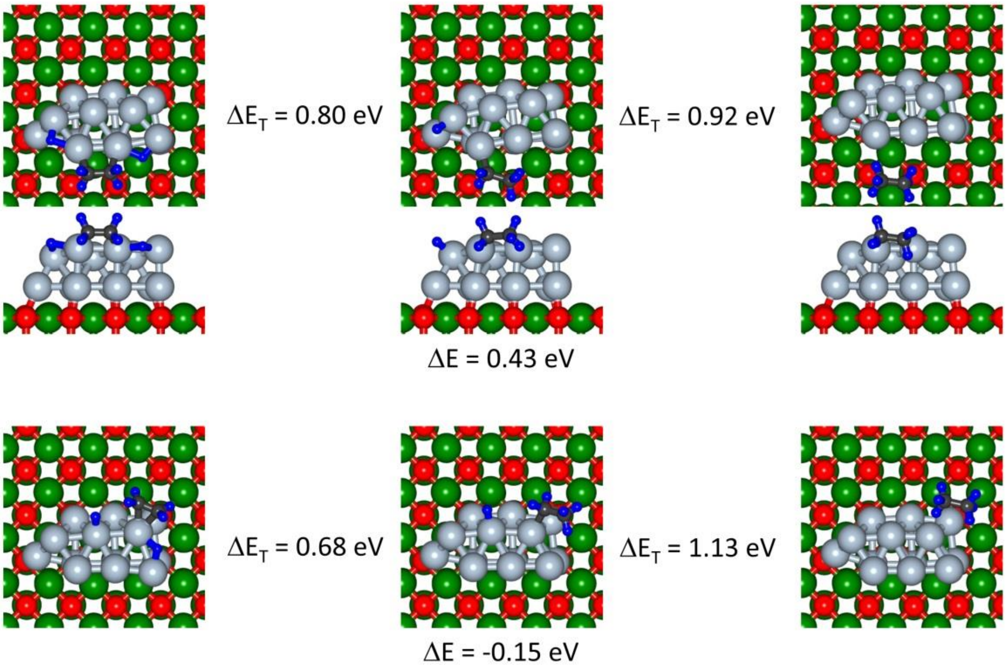

Supplementary Figure 30 | Higher-barrier pathways of ethylene hydrogenation on Pt_{13}/MgO. Higher-barrier (top-row, starting from di-σ bonded C_2H_4 coadsorbed with dissociated H_2) and high-barrier (bottom-row, starting from π-bonded C_2H_4 coadsorbed with dissociated H_2) pathways of ethylene hydrogenation on Pt_{13}/MgO.

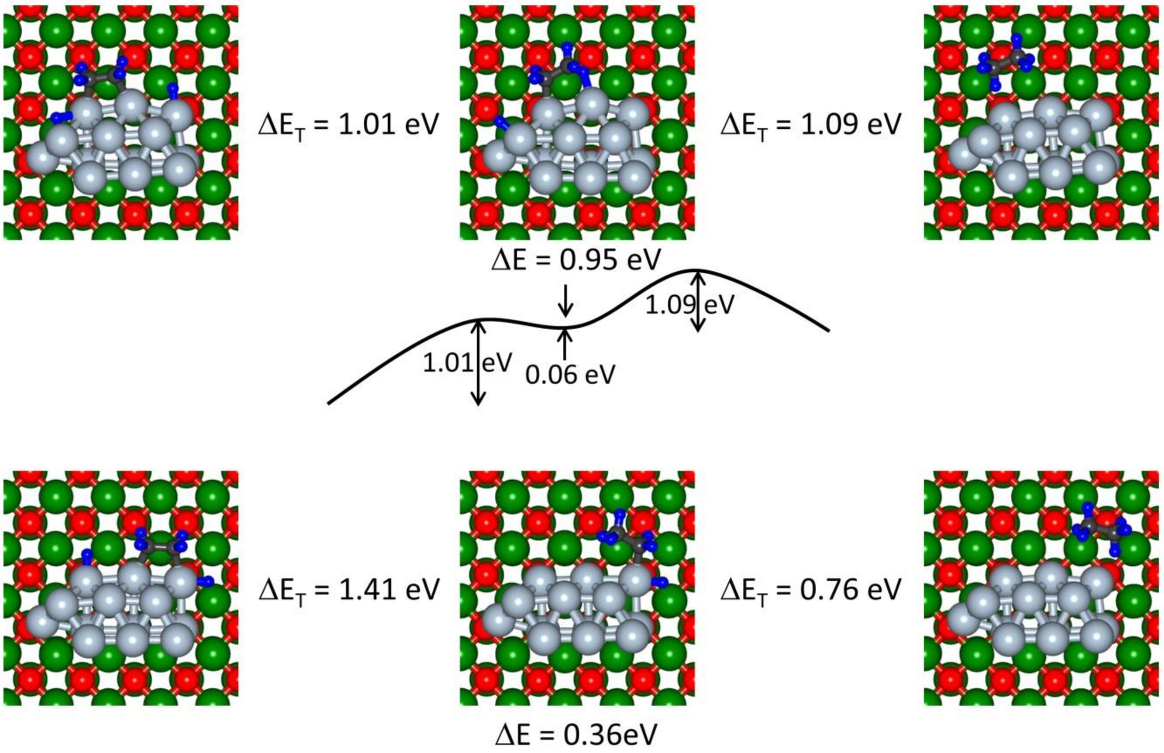

Supplementary Figure 31 | High-barrier pathways of ethylene hydrogenation on Pt_{13}/MgO.

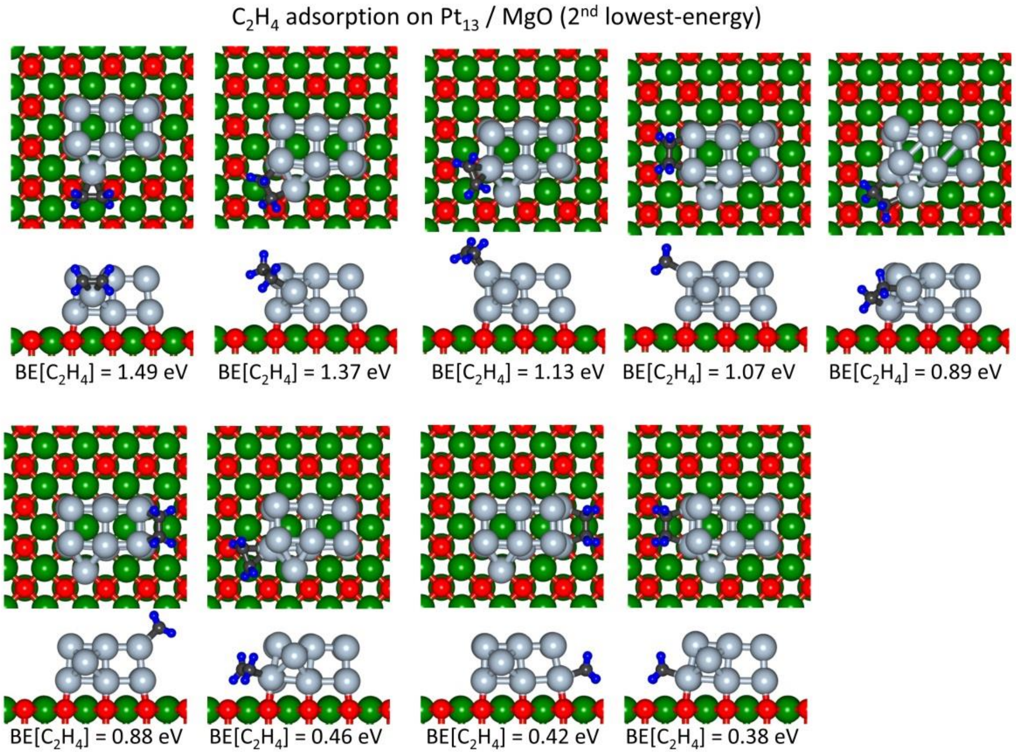

Supplementary Figure 32 | Binding sites of ethylene on the 2nd lowest isomer of the Pt_{13}/MgO system. see Supplementary Figure 11, isomer with ΔE = 0.14eV.

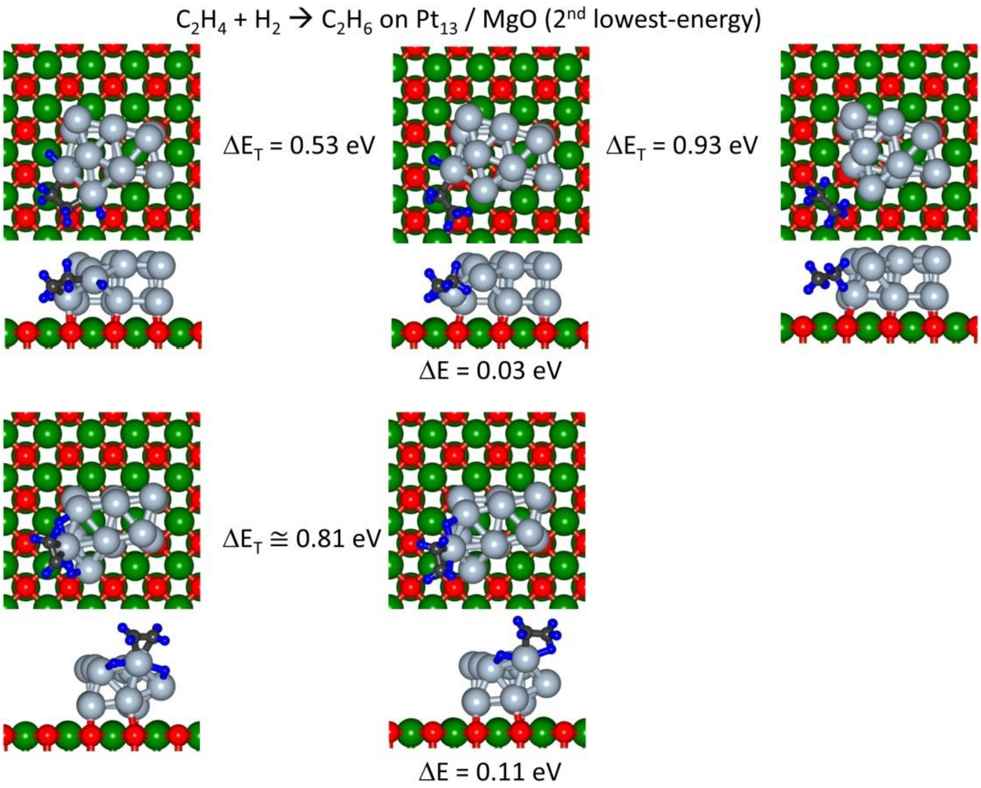

Supplementary Figure 33 | Higher-barrier pathways of ethylene hydrogenation on Pt_{13} (2nd isomer)/MgO.

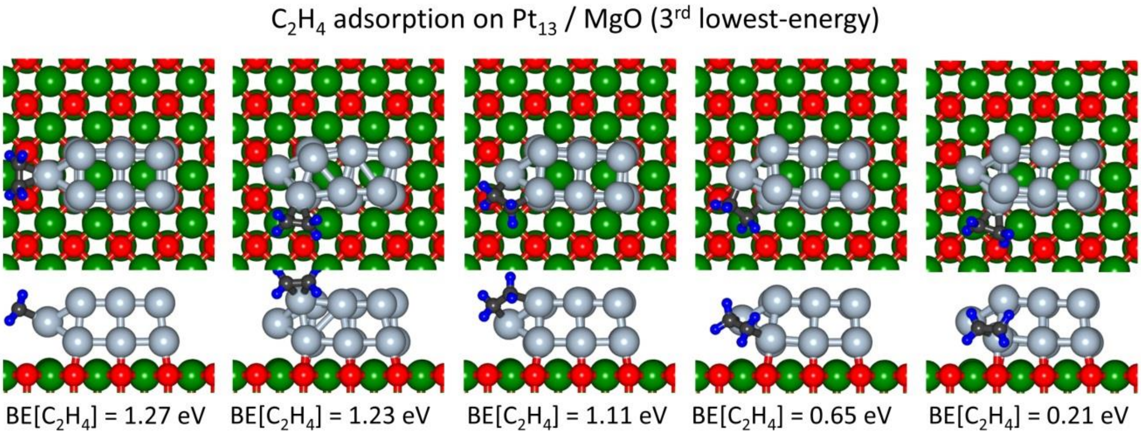

Supplementary Figure 34 | Binding sites of ethylene on the 3rd lowest isomer of the Pt_{13}/MgO system. See Supplementary Figure 11, isomer with $\Delta E = 0.23$eV.

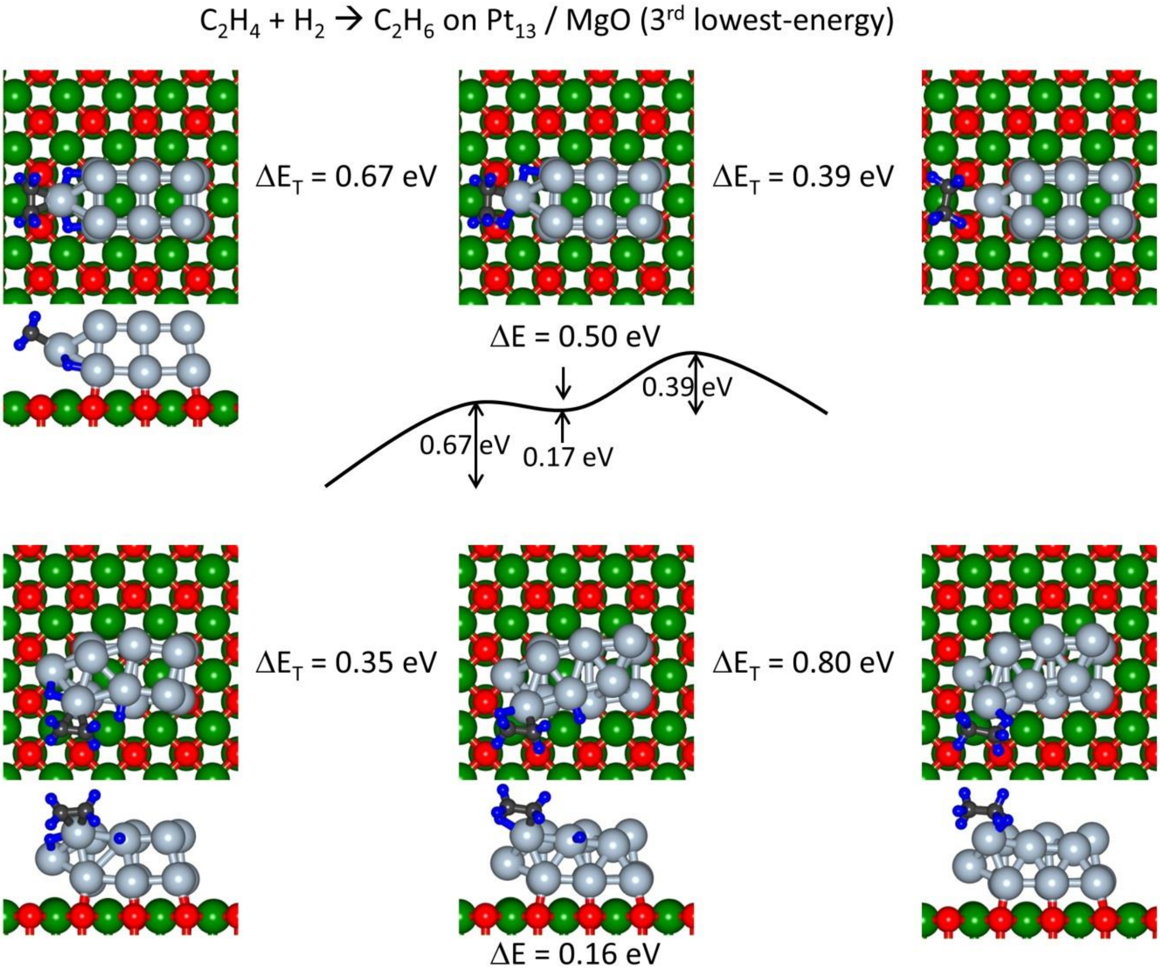

Supplementary Figure 35 | Low and hih energy barrier for ethelene hydrogenation on isomeric Pt_{13}/MgO. Low energy pathway (top row) and higher energy pathway (bottom row,) both starting from π-bonded C_2H_4 coadsorbed with dissociated H_2 on the 3rd-lowest isomer of the Pt_{13}/MgO system.

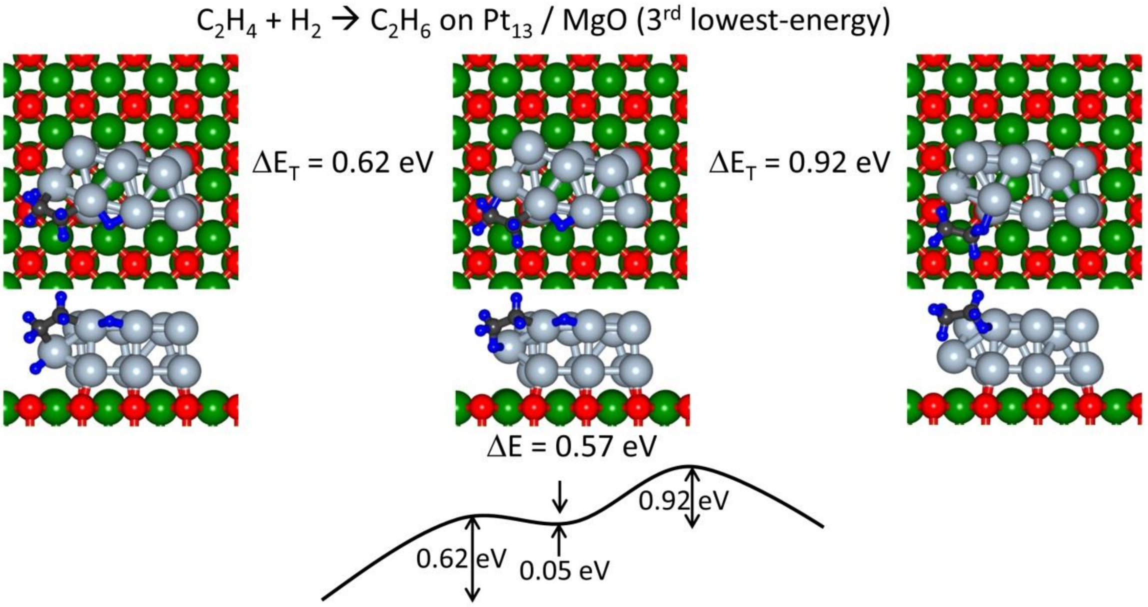

Supplementary Figure 36 | Higher energy pathway, starting from ethylene di-σ bonded with coadsorbed dissociated H_2. The path way is calculated for the 3rd-lowest isomer of the Pt_{13}/MgO system (compare to the high energy barrier patway calculated for the π-bonded C_2H_4 shown in Supplementary Figure 35).

Supplementary Note 1 | Structure insensitivity/sensitivity of ethylene hydrogenation

The Horiuti-Polanyi (HP) mechanism begins with hydrogen dissociation on the metal catalyst surface and following alkene (ethylene) adsorption, hydrogen additions occur in two subsequent steps: first, converting the alkene (C_2H_4) into an adsorbed alkyl intermediate (ethyl, $-CH_2CH_3$) followed by a second hydrogenation to form the alkane (ethane, C_2H_6) product which desorbs from the surface. Vibrational spectroscopy on single crystal surfaces revealed two strongly bound species present during the catalytic reaction on Pt(111) and Pt (100) surfaces (although with different relative concentrations)[1]: (i) di-σ ethylene where the carbon atoms of the adsorbed C_2H_4 molecule are each σ-bonded (in near sp^3 hybridization) to neighboring Pt surface atoms (see Fig. 2 in main article), and (ii) an ethylidyne (≡C-CH_3) molecule which may form through several pathways (for a recent discussion see [2]). Among these we note a favored reaction scheme that starts from adsorbed di-σ ethylene and proceeds through hydrogenation-dehydrogenation reactions with formation of the ethyl molecule (via hydrogenation) followed by two dehydrogenations: the first one converting CH_2CH_3 to ethylidene ($CHCH_3$) and the second yielding ethylidyne. Both the di-σ ethylene and ethylidyne molecules occupy three-fold hollow surface sites (involving reorganization of the neighboring metal atoms); on Pt(111) the ethylidyne is readily formed at around 280 K, and both, it and di-σ adsorbed ethylene are found on *all* platinum surfaces as stagnant spectators (that is they are strongly bound and do not participate in the reaction being measured). Instead the structure-insensitive hydrogenation of ethylene (occurring at higher hydrogen pressure and temperature) involves as reaction intermediates a weakly-bound π-bonded ethylene (in a near sp^2 hybridization) and the "half-hydrogenated" ethyl (C_2H_5) molecule; for a proposed reaction scheme see Fig. 13 in[3].

Real catalysis typically entails highly dispersed small particles supported on metal oxides or other high-surface-area substrates, whereas the results that were reviewed above were all obtained from investigations on extended single crystal metal surfaces. To bridge the so called "material - gap" attention has been shifted over the past decade to investigations involving finite particles on solid supports [4,5]. It is pertinent to remark here that the intrinsic size-effect of platinum particles supported on amorphous alumina in the hydrogenation of ethylene has been previously addressed [6]. In this investigation it was found that the reaction on Pt particles larger than 1.7 nm was structure insensitive and a turn over frequency (TOF) maximum was found for a particle size of ~ 0.6 nm (containing 10-20 atoms), with a similar

result found for a Pt/SiO_2 system. From these results, it was suggested that underlying the apparent structure sensitivity at small sizes was "increased atom accessibility"; this interpretation was reached, in the absence of realistic quantitative estimates, based on analysis that considered rather idealized simple polyhedral models and heuristic arguments. Additionally, the above experiment has been carried out on polydispersed particle samples, and a deconvolution of the effect of the particle-size distribution could not be made unambiguously. Several studies on the catalytic properties of Pt particles have been reported in the past few years, [see refs. 11-18 in Ref [7]] finding structure insensitivity for ethylene hydrogenation on particles in the range of 1-11 nm, albeit using polydispersed particle samples.

To summarize: current opinion is that while the adsorption of ethylene is structure sensitive, the overall hydrogenation reaction is structure insensitive.

Supplementary Note 2 | The Dewar-Chatt-Duncanson model

In all cases (including hydrogenation of C_2H_4 on Pt(111)) the microscopic reaction mechanism has been found to follow a frontier orbital description that find its origins in an adaptation of the Dewar-Chatt-Duncanson (DCD) model; here the addition of a hydrogen atom to the adsorbed molecule is described as an agnostic process (a term used to refer specifically to situations in which a hydrogen atom is covalently bonded to both a carbon and a transition metal atom[8], with the change in the C-H distance affecting (increasing) the energy gap between the bonding (σ_{CH}) and antibonding (σ_{CH}^*) states that shift away from the Fermi level as the C-H distance reduces (or equivalently the Pt-H distance increases). At the top of the activation barrier, the interaction between the σ_{CH} and σ_{CH}^* orbitals with the s-, p- and d-electrons of the Pt clusters brings about orbital mixing that may be described in term of the DCD donation and back-donation terms, culminating in attachment of the transferred H atoms (initially bonded to the Pt cluster) to the adsorbed molecule.

Supplementary references

1 Cremer, P. S., Su, X., Shen, Y. R. & Somorjai, G. A. Ethylene Hydrogenation on Pt(111) Monitored in Situ at High Pressures Using Sum Frequency Generation. *J. Am. Chem. Soc.* **118**, 2942-2949 (1996).

2 Zhao, Z.-J., Moskaleva, L. V., Aleksandrov, H. A., Basaran, D. & Rösch, N. Ethylidyne Formation from Ethylene over Pt(111): A Mechanistic Study from First-Principle Calculations. *J. Phys. Chem. C* **114**, 12190-12201 (2010).

3 Somorjai, G. A. & McCrea, K. Roadmap for catalysis science in the 21st century: a personal view of building the future on past and present accomplishments. *Appl. Catal. A* **222**, 3-18 (2001).

4 Gao, F. & Goodman, D. W. M\odel Catalysts: Simulating the Complexities of Heterogeneous Catalysts. *Annual Review of Physical Chemistry* **63**, 265-286 (2012).

5 St.Clair, T. P. & Goodman, D. W. Metal nanoclusters supported on metal oxide thin films: bridging the materials gap. *Topics in Catalysis* **13**, 5-19 (2000).

6 Masson, A. *et al.* Intrinsic size effect of platinum particles supported on plasma-grown amorphous alumina in the hydrogenation of ethylene. *Surf. Sci.* **173**, 479-497 (1986).

7 Sapi, A. *et al.* Recovery of Pt Surfaces for Ethylene Hydrogenation-Based Active Site Determination. *Catal. Lett.* **144**, 1151--1158 (2014).

8 Brookhart, M. & Green, M. L. H. Carbon-hydrogen-transition metal bonds. *J. Organometall. Chem.* **250**, 395-408 (1983)
